# The effector AWR5 from the plant pathogen *Ralstonia solanacearum* is an inhibitor of the TOR signalling pathway

**DOI:** 10.1038/srep27058

**Published:** 2016-06-03

**Authors:** Crina Popa, Liang Li, Sergio Gil, Laura Tatjer, Keisuke Hashii, Mitsuaki Tabuchi, Núria S. Coll, Joaquín Ariño, Marc Valls

**Affiliations:** 1 Centre for Research in Agricultural Genomics (CSIC-IRTA-UAB-UB), Bellaterra, Catalonia, Spain; 2 Genetics Department, Universitat de Barcelona, Barcelona, Catalonia, Spain; 3 Institut de Biotecnologia i Biomedicina and Departament de Bioquímica i Biologia Molecular, Universitat Autònoma de Barcelona, Cerdanyola del Vallès, Catalonia, Spain; 4 Laboratory of Applied Molecular and Cell Biology, Kagawa University, Kagawa, Japan

## Abstract

Bacterial pathogens possess complex type III effector (T3E) repertoires that are translocated inside the host cells to cause disease. However, only a minor proportion of these effectors have been assigned a function. Here, we show that the T3E AWR5 from the phytopathogen *Ralstonia solanacearum* is an inhibitor of TOR, a central regulator in eukaryotes that controls the switch between cell growth and stress responses in response to nutrient availability. Heterologous expression of *AWR5* in yeast caused growth inhibition and autophagy induction coupled to massive transcriptomic changes, unmistakably reminiscent of TOR inhibition by rapamycin or nitrogen starvation. Detailed genetic analysis of these phenotypes in yeast, including suppression of AWR5-induced toxicity by mutation of *CDC55* and *TPD3*, encoding regulatory subunits of the PP2A phosphatase, indicated that AWR5 might exert its function by directly or indirectly inhibiting the TOR pathway upstream PP2A. We present evidence *in planta* that this T3E caused a decrease in TOR-regulated plant nitrate reductase activity and also that normal levels of TOR and the Cdc55 homologues in plants are required for *R. solanacearum* virulence. Our results suggest that the TOR pathway is a bona fide T3E target and further prove that yeast is a useful platform for T3E function characterisation.

Many bacterial pathogens use a type III secretion system (T3SS) to inject a suite of proteins inside the host cell^1^. These proteins are referred to as type III effectors (T3Es), and play a central role in bacterial survival and disease development^2^. T3Es manipulate host cell pathways by mimicking key host proteins or mediating changes in their subcellular localization, by targeting plant-specific transcription factors, by inhibiting translation and metabolic stress pathways or exploiting a specific form of host-mediated fatty acid modification^3^,^4^,^5^. The functional study of T3Es from phytopathogenic bacteria has raised a tremendous interest in the last years^6^,^7^. The number of T3Es identified is growing at a very fast pace as more bacterial genomes become available, revealing complex repertoires that feature internal redundancy, which complicates their study^6^. However, only in a few cases the function of this kind of effectors *in planta* has been identified.

Heterologous production in *Saccharomyces cerevisiae* has offered promising and effective strategies to characterize bacterial T3Es^8^. Seminal work with YopE showed that this T3E caused specific growth inhibition and cytoskeletal alteration, an activity conserved in yeast and mammalian cells^9^. Functional analyses of plant-associated T3E in yeast have revealed other effector-triggered phenotypes including cell death, suppression of apoptosis or perturbation of host cellular processes, such as MAPK signalling or sphingolipid synthesis^10^,^11^,^12^. All these findings strengthen the premise that many bacterial T3E target universal eukaryotic processes so that *S. cerevisiae* can be exploited to elucidate their molecular function and to investigate target-effector interactions^8^,^13^.

The TOR complex 1 (TORC1) is a central regulator of cell growth in response to nutrient availability and stress conditions by controlling diverse cellular processes, including transcriptional activation, ribosome biogenesis or autophagy^14^ (Fig. 1a). This complex contains the Tor1 or Tor2 protein kinases and can be inhibited by the drug rapamycin. In yeast, TORC1 acts by controlling three major cell components: the kinase Sch9, Tap42 and its associated phosphatases and the ATG1 complex^14^,^15^. Thus, TORC1 modulates nitrogen catabolite repression and diverse stress responses by controlling the activity of several phosphatases, such as protein phosphatase 2A (PP2A) or Sit 4, often by modifying their interaction with regulatory subunits (Fig. 1a^15^).

*Ralstonia solanacearum* is emerging as a model system to study plant-pathogen molecular interactions and T3E function^16^. This soil-borne bacterium is the causing agent of bacterial wilt, a disease caused when the bacterium growing in plant extracellular spaces (apoplast) infects the xylem vessels, where it multiplies extensively and blocks water flow^17^. *R. solanacearum* has been ranked as the second most important bacterial plant pathogen^18^, due to its high persistence and wide geographical distribution and host range, as it infects more than 200 plant species, including important agricultural crops such as tomato and potato^19^. Of more than 70 T3Es identified in the reference strain GMI1000, only for two of them a defined role *in planta* has been assigned^16^. AWRs (named after a conserved alanine-tryptophan-arginine tryad and also called RipAs) are one of the multigenic families of T3Es conserved in all *R. solanacearum* strains^17^, with orthologues in other bacterial pathogens such as *Xanthomonas* strains, *Acidovorax avenae* or *Burkholderia* spp.^20^. A low protein similarity has also been described between AWRs and the *Xanthomonas oryzae pv. oryzae* effector XopZ, which was shown to be involved in virulence and suppression of host basal defence^21^. Translocation assays have proven AWRs as *bona fide R. solanacearum* type III secreted effectors^20^,^22^,^23^. However, sequence information on AWR proteins gives no clue on their putative function. In a previous study, we showed that the AWR T3E family collectively contributes to *R. solanacearum* virulence, as a mutant bacterium devoid of all AWR multiplies 50-fold less than the wild-type strain on eggplant and tomato plants. Functional analysis of AWRs also demonstrated that their expression in different plant species triggers varying defence responses^20^. Functional analyses for each AWR showed that AWR5 had an important contribution in virulence and also caused the most dramatic plant responses. In addition, we have recently found that *awr5* is one of the most highly expressed genes when *R. solanacearum* grows inside the plant host (Marina Puigvert, unpublished results). Association genetics combining genomic data from *R. solanacearum* strains and their pathogenicity on eggplant, pepper and tomato accessions identified AWR5 amongst the three T3Es highly associated to virulence^24^.

In this work we take advantage of the yeast system to characterize AWR5 function. Heterologous expression of *AWR5* in *S. cerevisiae* resulted in dramatic growth inhibition of yeast cells. We show that this effect on yeast growth is caused by inhibition of the central regulatory TOR pathway. Importantly, AWR5 impact on the TOR pathway is conserved in both yeast and plants, revealing a previously unknown T3E mode of action maintained in evolutionary distant organisms. Moreover, our work further validates yeast as an excellent platform to uncover T3E function.

## Results

### Expression of the *R. solanacearum awr* type III effector family in yeast causes growth inhibition

To investigate the function of the AWR bacterial effectors in eukaryotic cells, we expressed the five *awr* genes from *R. solanacearum* GMI1000 in *S. cerevisiae.* In a first step, *awr*s were cloned in the high-copy-number vector pAG426GAL, where they are transcribed from the strong galactose-inducible *GAL1* promoter. The resulting plasmids were introduced in yeast and the transformed strains grown overnight, then serially diluted and plated either in repressing media (glucose) or inducing media (galactose). It was observed that, except for AWR4, these effectors inhibited growth to different extents, as observed by the inability to form macroscopic colonies on inducing media (Supplementary Fig. S1). AWR1, 2, 3 and 5 caused a strong toxicity upon induction, but AWR5 showed the most dramatic effect, inhibiting yeast growth even in non-inducing conditions. The phenotype seemed specific for AWR effectors, as it was not observed when a control gene (GFP) was expressed (Supplementary Fig. S1). The full-length AWR5 protein was required for functionality, as expression of split variants of AWR5 (N-terminal or C-terminal halves, or the central region) did not cause toxicity (Supplementary Fig. S1).

**Figure 1:**
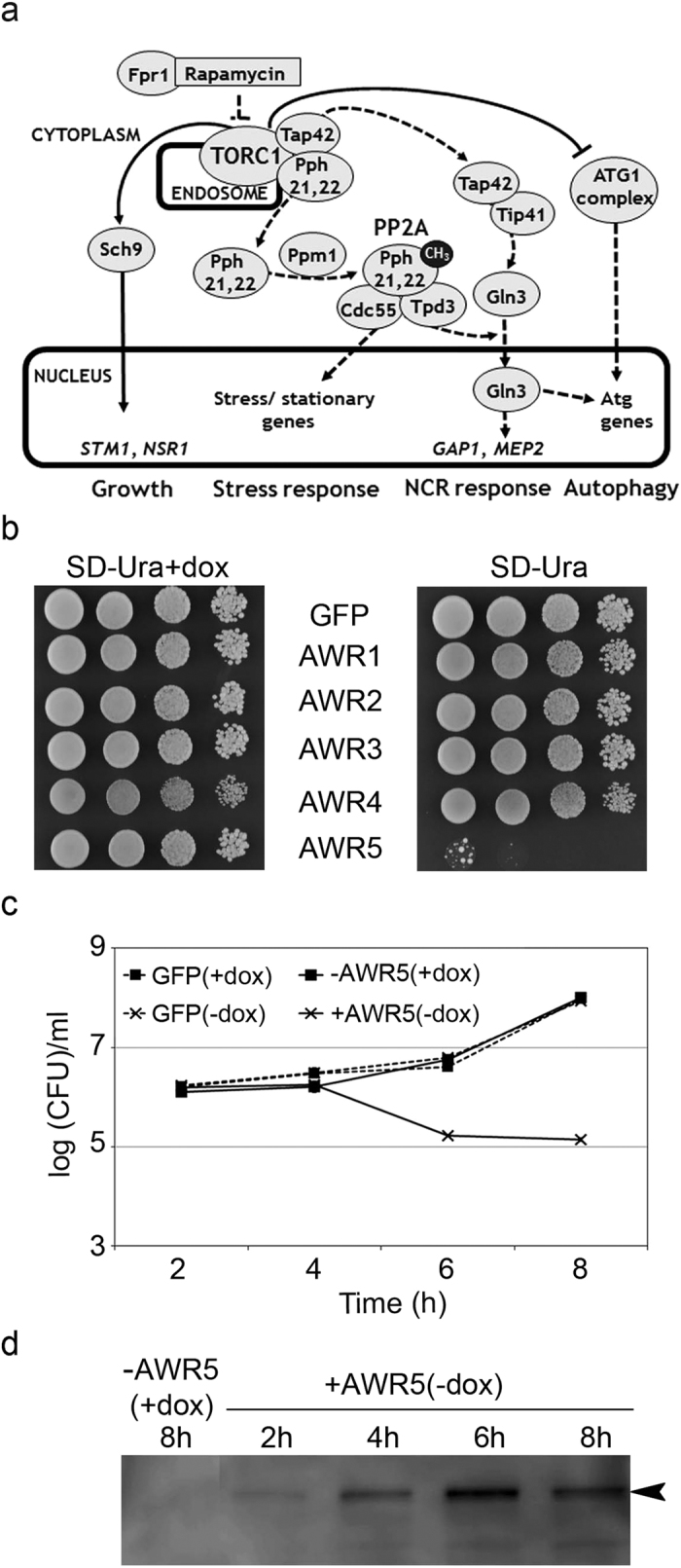
Expression of *awr5* effector inhibits yeast growth. (**a**) Schematic view of the *Saccharomyces cerevisiae* TORC1-regulated pathways. The TORC1 complex is a central growth regulator, controlling the balance between growth and quiescence. Continuous and dotted lines represent, respectively, signaling events regulated by active and inactive TORC1. (**b**) Growth on solid medium of yeast strains expressing *awr* effectors. Yeast strains bearing *awr* genes fused to GFP tag or a GFP control were subjected to serial 10-fold dilutions and spotted onto solid SD-Ura+doxycycline (repressing medium) and SD-Ura (inducing medium). Photographs were taken after 2 days of growth. (**c**) Growth kinetics in liquid medium of yeast cells harboring *awr5* or a GFP control. Yeast cells harboring *awr5* or a GFP control were grown in SD-Ura+dox (−AWR5) and SD-Ura (+AWR5) liquid media and dispersed on SD-Ura+dox plates. The logarithm of colony forming units (CFU) per ml is shown over time. Error bars indicate standard errors for 2 biological replicates. (**d**) Immunoblot analysis of AWR5 protein levels. Total protein was extracted from cultures shown in Fig. 1c and immunoblotted using an anti-GFP antibody. The black arrowhead indicates AWR5-GFP protein. All experiments were performed at least three times, with similar results.

To evaluate the phenotype in more physiological conditions and ensure construct stability and tight control of effector transcription, we integrated the bacterial genes in the yeast genome under the control of a repressible Tet-Off promoter. When the resulting strains bearing *awr*s or a control GUS gene were plated in the absence of the repressor doxycycline, only expression of *awr5* reproduced the dramatic growth arrest (Fig. 1b). The absence of toxicity for AWR1, 2 and 4 could not be attributed to a lack of expression, as the full-length proteins were readily detected in yeast cells (Supplementary Fig. S2). Thus, we concentrated on the characterization of the growth inhibition caused by *awr5* expression.

### Characterization of the AWR5-dependent growth inhibition phenotype

Yeast growth inhibition was also apparent upon AWR5 production in liquid cultures as indicated by a rapid stagnation of cell density over time (not shown) and a clear decrease in the number of growing cells (Fig. 1c). Growth inhibition kinetics paralleled with an increase in *awr5* RNA (Supplementary Fig. S3) and protein levels (Fig. 1d). Microscopic observation of strains producing AWR5 revealed the presence of budding cells at similar proportions to cells not producing the bacterial effector (Supplementary Fig. S4a). Thus, it could be ruled out that this protein specifically alters the cell cycle.

Expression of *awr5* caused strong growth inhibition but not cell death, as deduced from methylene blue staining of cells bearing *awr5* in the absence of doxycycline (Supplementary Fig. S4a) and from counting of viable cells able to form colonies after 6 h of *awr5* expression (Supplementary Fig. S4b). Similarly, growth arrest in cells expressing *awr5* was not likely caused by defects in cell wall construction leading to cell lysis, since it was not eliminated by osmotic stabilization with 10% sorbitol (Supplementary Fig. S5). In contrast, determination of cell size upon expression of *awr5* showed significant changes, visible after 8 h of induction, with AWR5-producing cells showing an average diameter of 4.96 ± 0.03 μm, while that of non-expressing cells was over 5.3 ± 0.06 μm (Supplementary Fig. S4c).

Previous reports studying effectors from *Pseudomonas syringae* or *Xanthomonas euvesicatoria* had shown that some of them caused growth arrest when yeast was forced to respire^10^,^11^. To verify if respiration affected AWR5 toxicity in yeast, we grew serial dilutions of the strain producing this protein or a control gene (β-glucuronidase, GUS) onto solid medium containing the non-fermentable carbon sources ethanol and glycerol. As observed in Supplementary Fig. S5, the toxic effect due to AWR5 was maintained under these conditions.

In summary, we established that production of the full-length AWR5 protein in yeast targeted a cellular process leading to growth inhibition and decreased cell size, but not involving an evident cell cycle arrest or cell death.

### Expression of *awr5* mimics the transcriptional changes induced by the TORC1 inhibitor rapamycin

To understand the molecular basis of *awr5* toxicity in yeast and to highlight putative functional targets, we considered the identification of possible changes at the mRNA level caused by expression of the effector. To this end, we carried out a genome-wide transcriptomic analysis using DNA microarrays in yeast cells with *awr5* expression induced for 2, 4 and 6 h. This time-course was selected according to the previously characterized growth effect (Fig. 1c). DNA microarray analysis yielded 3763 genes with valid data for all 3 time-points. We observed that induction of *awr5* expression produced relevant time-dependent changes in the transcriptomic profile that, in most cases, could be observed after 4 and 6 h of induction. The mRNA level of 766 genes was modified at least 2-fold, with 319 genes induced and 447 repressed. The functional assignment of induced genes revealed a striking excess of genes subjected to nitrogen catabolite repression (NCR)^25^, such as *MEP2*, *GAP1*, *DAL5*, *CPS1* or *DUR1,2*, whereas among the repressed genes there was a vast excess of genes encoding ribosomal proteins or involved in ribosome biogenesis. This profile was reminiscent of that reported by several laboratories for inhibition of the TORC1 pathway^15^.

**Figure 2:**
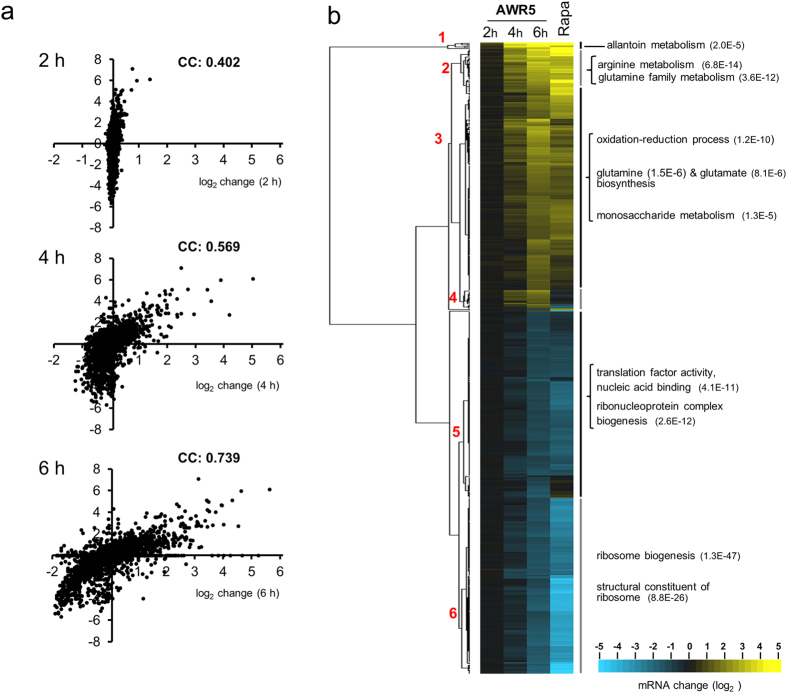
Expression of bacterial *awr5* in yeast mimics the transcriptomic changes caused by inhibition of the TORC1 pathway. (**a**) Changes in mRNA levels caused by expression of *awr5* (X-axis, log2 space) for the set of 2774 genes with valid data for all three time-points were plotted against the corresponding values after 1 h treatment with 200 ng/ml rapamycin (Y axis, log2 space). “CC” figures indicate the calculated correlation coefficient among both sets of data for each time-point. (**b**) The set of 596 genes presenting at least 2-fold changes in mRNA levels upon expression of *awr5* and with valid data for the rapamycin treatment were clustered (Euclidean distance, average linkage) using Cluster 3.0 software^57^ and are represented with the Java Treeview software, version 1.1.6r4^58^. Numbers in red denote selected clusters referred to in the main text and number between parentheses designate the p-value for the indicated GO annotations.

We took advantage of recent work in our laboratory in which the transcriptomic profile in response to 1 h of exposure to rapamycin had been generated^26^. Combination of this data with that obtained here after *awr5* expression yielded 2774 genes with expression information in both conditions. Figure 2a shows the correspondence between changes produced in response to *awr5* with those caused by rapamycin. It can be observed that whereas the correlation is relatively poor shortly after *awr5* induction (correlation coefficient = 0.402), the similarity between both responses becomes evident after 4 h and, particularly, after 6 h of *awr5* induction (correlation coefficients 0.569 and 0.739, respectively). We then selected among the 766 genes whose expression changed at least 2-fold those with data for the rapamycin treatment (596 genes) and subjected this set of genes to clustering analysis. Figure 2b clearly documents that the time-dependent transcriptional response to expression of *awr5* matches that provoked by rapamycin treatment (correlation coefficient of 0.872 when compared with *awr5* data after 6 h of expression). It can be observed that clusters 1 and 2 -and to some extent also cluster 3- are enriched in induced genes related to metabolism of nitrogen (mostly amino acids), whereas regarding the repressed genes, cluster 5 includes genes involved in translation and cluster 6 is enriched in genes encoding ribosomal proteins or members of the RiBi (ribosome biogenesis) regulon. All these results indicate that expression of bacterial *awr5* in yeast triggers a response that mimics the inhibition of the TORC1 pathway.

**Figure 3:**
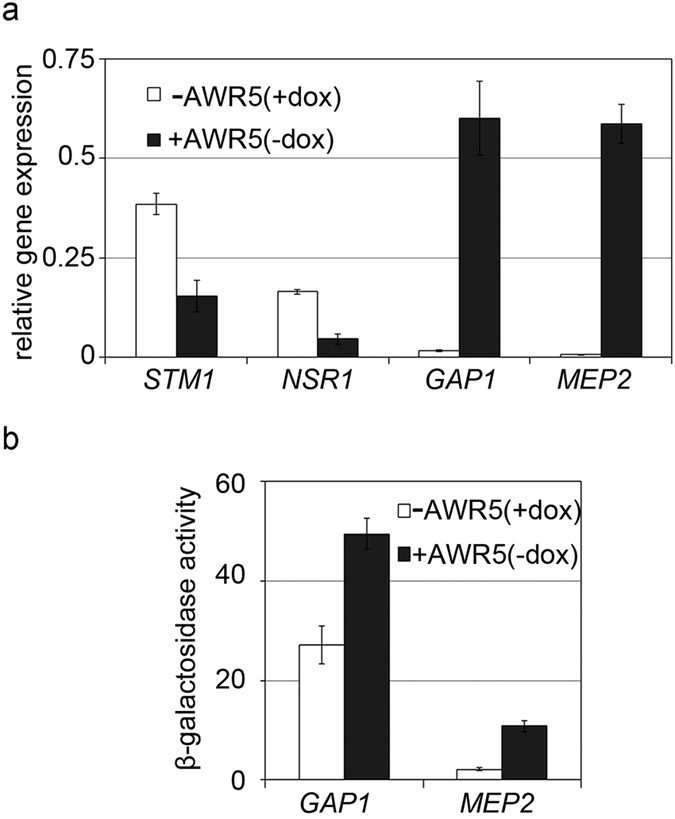
Transcriptional response of TORC1-related genes to *awr5* expression. (**a**) qRT-PCR experiments showing relative gene expression of TORC1 downstream targets. Gene expression of nitrogen catabolite repression (NCR)-sensitive *GAP1* and *MEP2* and ribosomal biogenesis *STM1* and *NSR1* genes was tested in yeast strains expressing *awr5* (+AWR5 (−dox)) 6 hours after induction. Error bars represent standard errors from 2 biological replicates. (**b**) β-galactosidase activities from yeast cells bearing *awr5*. Promoter activities of *GAP1* and *MEP2* were determined 6 hours after growth in SD-Ura+dox (−AWR5) and SD-Ura (+AWR5). Data represent the means and standard errors of 4 independent clones. All assays were repeated at least twice with similar results.

These transcriptomic data were validated by performing quantitative RT-PCR analysis on a subset of genes from different TORC1-regulated pathways, which showed altered expression levels in response to *awr5* (Fig. 3b). As expected, *awr5* expression resulted in a decrease of the levels of the TOR-activated *STM1* and *NSR1* genes, which are involved in yeast growth^27^,^28^. In contrast, the levels of the TOR-repressed *GAP1* and *MEP2*, which control nitrogen catabolite repression^29^, increased in response to *awr5* expression. Similar results were obtained when promoter activity was measured using fusions to the β-galactosidase reporter: *awr5* expression resulted in increased *GAP1* and *MEP2* promoter output (Fig. 3b).

### Mutations in two genes involved in the TORC1 pathway rescue the yeast growth inhibition caused by AWR5

**Figure 4:**
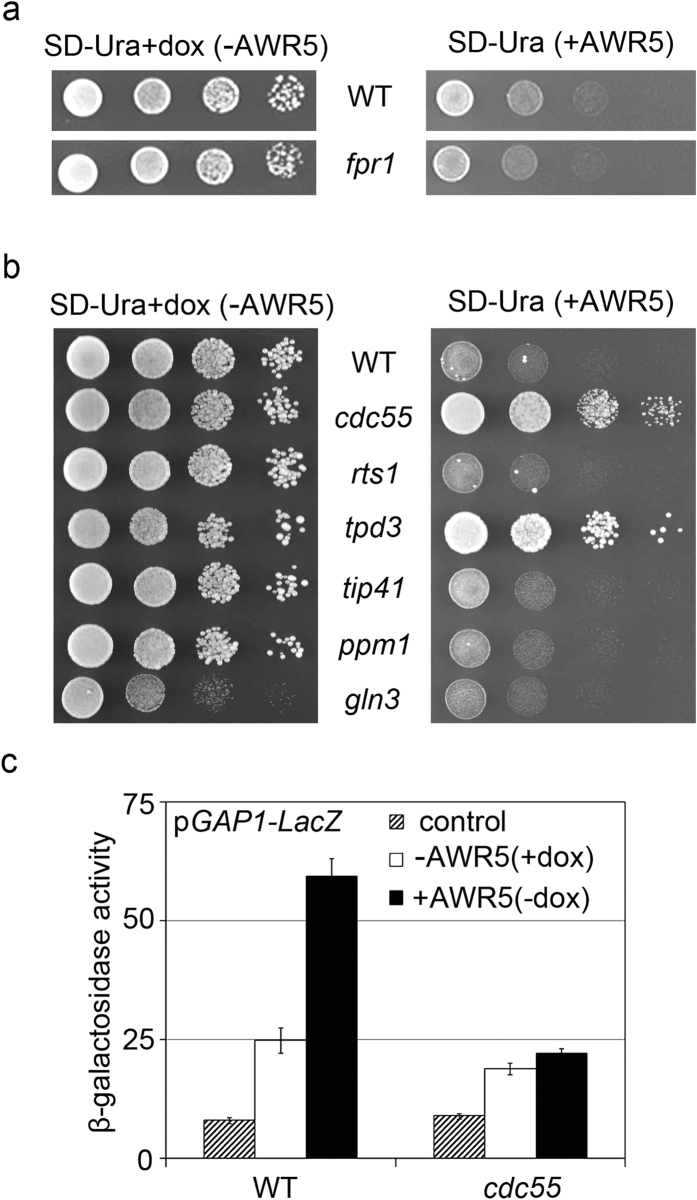
c*dc55* and *tpd3* mutations affecting PP2A protein phosphatase activity suppress AWR5-induced yeast growth inhibition. (**a**) Growth on solid medium of control (WT) and an *fpr1* mutant carrying *awr5* under the control of a Tet-Off promoter. Serial 10-fold dilutions were spotted onto solid SD-Ura+doxycycline (−AWR5) and SD-Ura (+AWR5). (**b**) Growth on solid medium of control (WT) and TORC1-related yeast mutants containing plasmid carrying *awr5*. Serial 10-fold dilutions were spotted onto solid SD-Ura+doxycycline (−AWR5) and SD-Ura (+AWR5). Photographs were taken after 3 days of growth. (**c**) *GAP1* promoter activity from plasmid *pGAP1-LacZ* in wild-type (WT) and mutant *cdc55* yeast cells bearing *awr5* or a control gene (*GFP*). β-galactosidase activity was measured 6 hours after growth in SD-Ura+dox (−AWR5) and SD-Ura (+AWR5). Values represent the means and standard errors of 4 independent clones. All experiments were performed three times with similar results.

Since AWR5 mimicked rapamycin treatment in yeast, we tested whether disruption of *FPR1*-encoding the rapamycin-binding protein Fpr1 that inhibits the TORC1 kinase in the presence of rapamycin^15^ rescued the AWR5-triggered phenotype. Growth inhibition caused by AWR5 was maintained in the *fpr1* strain (Fig. 4a), indicating that the bacterial effector acts on TORC1 through a different mechanism than rapamycin.

In order to ascertain which point of the TOR-controlled pathways was targeted by AWR5 we analysed yeast strains with altered levels of different genes mediating TORC1 signalling. Interestingly, the strains mutated in the PP2A regulatory or scaffold subunits *cdc55* or *tpd3* did not show AWR5-triggered growth inhibition (Fig. 4b). This indicated that these PP2A subunits are essential for AWR5 to cause its phenotype. These results were also corroborated by testing promoter activity of *GAP1* fused to the β-galactosidase reporter in wild type and *cdc55* mutant strains. Our results clearly showed that *CDC55* was required for the increase in *GAP1* promoter activity that occurs in response to *awr5* expression (Fig. 4c).

On the contrary, AWR5 did not seem to target the PP2A catalytic subunit, since AWR5-mediated growth inhibition could not be rescued by overexpression or conditional mutation of the two redundant genes (*pph21, 22*) encoding this subunit (Supplementary Fig. S6a,b). Any other mutant (*rts1*, *tip41*, *ppm1* and *gln3*) or overexpressor (*SIT4*) in genes related to signalling through the TORC1 pathway that we tested did not show reversion of AWR5-mediated growth inhibition. However, we could not detect interaction between Cdc55 or Tpd3 and AWR5 in yeast using co-immunoprecipitation (Supplementary Fig. S7a,b). Although the transcription profile was specifically compatible with TORC1 inhibition, we checked whether AWR5 had any impact on TORC2. As shown in supplementary Fig. S8, AWR5 does not interfere with TORC2, because a dominant active *ypk2* mutant (one of the major downstream components of the TORC2 pathway) did not rescue growth inhibition caused by AWR5 (Fig. S8a) and expression of the effector did not alter the actin cytoskeleton, a target of the TORC2 pathway (Fig. S8b). In addition, AWR5 also did not co-immunoprecipitate with the Lst8, a shared component of TORC1 and TORC2^30^ (Supplementary Fig. S7c).

**Figure 5:**
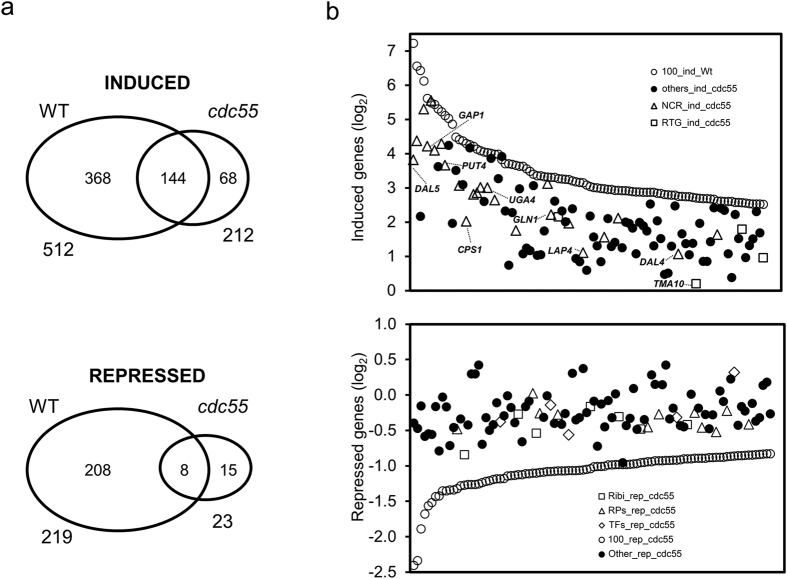
Mutation of *CDC55* greatly attenuates AWR5 impact on the yeast transcriptional profile. (**a**) Venn diagram showing the number of genes whose expression was considered to be induced (top) or repressed (bottom) by expression of AWR5 in wild-type and *cdc55* cells for a set of 5732 genes with valid data for both strains. (**b**) Plots of the log2 values for the changes in the level of expression induced by expression of AWR5 in both wild-type (open circles) and *cdc55* strains for the 100 most upregulated (top) and 100 most downregulated (bottom) genes in the wild-type strain (open circles). Symbols for the expression values for the *cdc55* strain are depicted as follows. For the induced genes: open triangles, the NCR family, as defined previously^25^; the RTG group (open squares) comprises the genes described as documented targets for the Rtg1 or Rtg3 transcription factors as defined in^59^. Genes not included in these categories are designated as “others” (closed circles). The genes downregulated in the wild-type strain are classified into one of three possible families: Ribi regulon (open squares), ribosomal proteins (open triangles), protein translation (open diamonds), and others (closed circles), as defined in^59^.

To determine whether Cdc55 was required for downstream AWR5-mediated responses, we carried out a new transcriptomic analysis, in this case by direct sequencing of RNAs (RNA-seq) in wild type and *cdc55* cells expressing *awr5* for 6 h. Analysis of the wild type strain showed a response congruent with that observed previously using DNA microarrays, with a correlation coefficient of 0.63 in the genes detected as induced by both methodologies (Supplementary Fig. S9). In addition, among the top 25 most induced genes detected by microarray analysis, 13 were also ranked as such by RNA-seq. Comparison of the profiles of the wild type and the *cdc55* strains after 6 h of *awr5* induction showed that mutation in *CDC55* dramatically attenuated the transcriptomic effects caused by *awr5* expression. As illustrated in Fig. 5a, 512 genes were induced in the wild type strain upon *awr5* expression and only 212 in the *cdc55* strain (of which only 144 were also induced in wild type cells). This effect was particularly evident in repressed genes, since the *cdc55* mutation affected almost 90% of the genes repressed by *awr5* expression in the wild type strain. The attenuation of the transcriptional response to AWR5 could clearly be observed by plotting the 100 genes showing highest induction (Fig. 5b, upper panel) or repression (Fig. 5b, lower panel) in wild-type cells and comparing to their expression in *cdc55* cells.

It was apparent that many of the highly induced genes in response to AWR5 expression, which belong to the NCR and the mitochondrial retrograde pathways, decreased their expression in the absence of the regulatory subunit of PP2A. Indeed, 26 out of 28 NCR and *RTG* genes^31^ ranking as top 100 induced decreased their expression more than 50% in *cdc55* cells. Similarly, a significant number of genes whose expression was decreased in response to AWR5 were clearly no longer repressed in *cdc55* cells. However, the effect was not homogeneous. For instance the transcripts showing little or no change in *awr5*-induced repression upon deletion of *CDC55* are largely enriched in genes involved in ribosome biogenesis and rRNA processing (Supplementary Fig. S10). This could be expected, as TOR-regulated expression of these genes is mostly PP2A-independent.

Taken together, these results indicate that the inability to form PP2A complexes containing Cdc55 not only neutralizes the severe growth defect caused by expression of *awr5*, but also substantially minimizes the transcriptional alterations derived from such expression. These data further supported the notion that the PP2A complex might mediate the phenotype caused by the AWR5 effector.

### *awr5* expression constitutively activates autophagy

**Figure 6:**
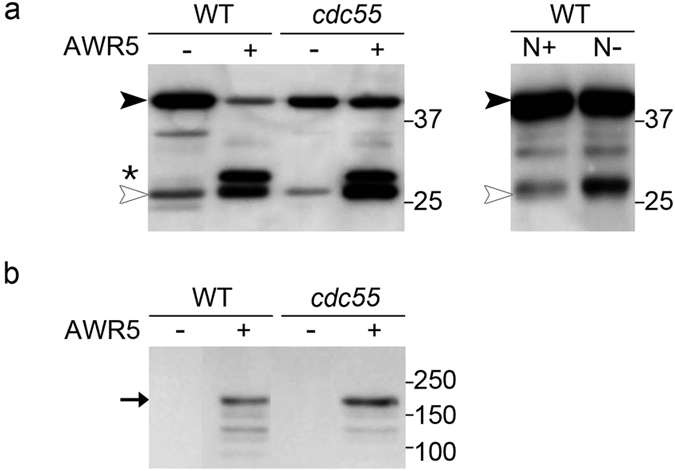
a*wr5* expression induces constitutive autophagy, independently of Cdc55-PP2A activity. (**a**) Immunodetection of GFP-ATG8 processing in wild-type and mutant *cdc55* yeast strains expressing *awr5*. Wild-type (WT) and mutant *cdc55* yeast cells bearing *awr5* gene were grown in SD-Ura+dox (−AWR5) and SD-Ura (+AWR5). Total protein extracts were immunoblotted using anti-GFP antibody. The black and the empty arrowhead indicate, respectively, GFP-ATG8 fusion protein and cleaved GFP. The asterisk denotes a degradation product of AWR5-GFP protein. (**b**) Wild-type cells carrying GFP-ATG8 grown in nitrogen-rich (N+) or nitrogen-depleted (N−) medium were used as a control of GFP-ATG8 processing and induction of autophagy in N− conditions. (**c**) AWR5 protein levels in wild-type and mutant *cdc55* yeast cells. Total protein was extracted and immunoblotted using anti-GFP antibody. The black arrow indicates AWR5-GFP protein. All experiments were performed at least three times, with similar results.

It is known that TORC1 regulates autophagy in yeast via inhibition of the ATG1 complex (Fig. 1a and^32^). Our microarray data showed that expression of *awr5* increased the expression of diverse autophagy genes, such as *ATG8* or *ATG14*, which indicates activation of this process. In order to confirm whether autophagy was affected by *awr5* expression, autophagic flux was monitored in yeast cells constitutively expressing *GFP-ATG8* (Fig. 6). Proteolysis of GFP-ATG8 in the vacuole during autophagy results in the accumulation of the GFP moiety. Hence, detection of free GFP levels by western blot analysis can be used as readout of the autophagic rate^33^. Expression of *awr5* led to a dramatic accumulation of GFP in yeast cells, indicating an increased autophagic flux (Fig. 6a). As a control, we subjected yeast cells to nitrogen starvation, which resulted, as expected, in an increase of free GFP levels (Fig. 6b). Interestingly, free GFP levels in *awr5*-expressing cells relative to GFP-ATG8 were higher than in nitrogen-starved cells, indicating that AWR5 expression induces autophagy more potently than nitrogen starvation does. Next, we tested whether Cdc55 was involved in AWR5-triggered autophagy in yeast. Although GFP-ATG8 levels were slightly higher in *cdc55* mutant cells expressing *awr5*, autophagy was similarly induced in both strains (Fig. 6a). *awr5* expression was analysed and similar levels were detected in wild type and *cdc55* mutant cells (Fig. 6c). These findings indicated that AWR5-mediated autophagy induction occurs independently of Cdc55 in yeast.

### AWR5 alters the TOR pathway in plants

**Figure 7:**
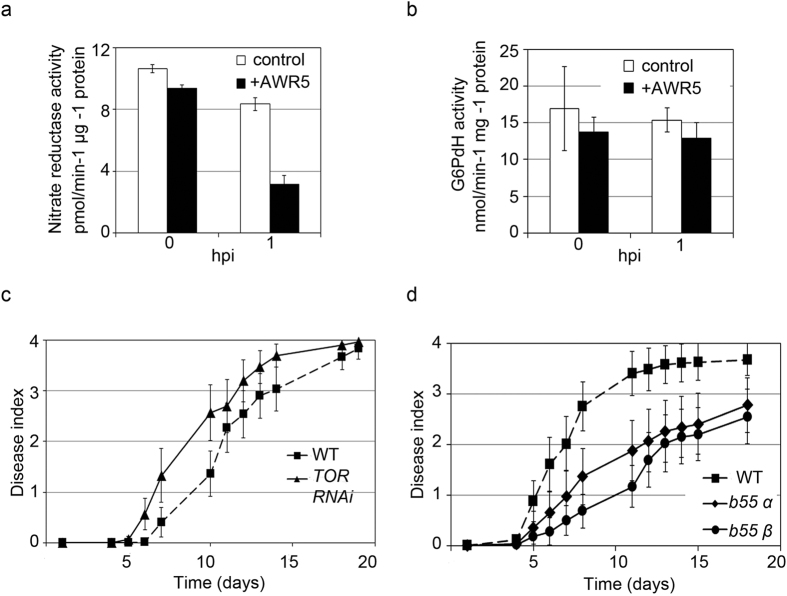
Interplay between AWR5 and TOR *in planta*. Effect of *awr5* transient expression on (**a**) nitrate reductase (NR) activity or (**b**) glucose-6-phosphate dehydrogenase (G6PdH) in *Nicotiana benthamiana*. Full leaves of *N. benthamiana* were agroinfiltrated with constructs bearing *awr5* or a control gene (*GUS*). Total protein extracts were used to determine NR and G6PdH activity at 0 and 1 hours post-estradiol induction (hpi). Error bars indicate standard errors of 2 biological replicates for NR and 3 for G6PdH. TOR (**c**) and its signalling component B55 (**d**) are involved in plant defence responses against *R. solanacearum* invasion. Five-week old plants grown in Jiffy pots were inoculated with *R. solanacearum* GMI1000 at an OD_600_ = 0.1 and wilting symptoms were recorded over time according to a disease index scale (0: no wilting, 1: 25% wilted leaves, 2: 50%, 3: 75%, 4: death). The experiment was repeated twice using at least 20 plants in each. Error bars indicate standard errors.

Since heterologous expression of a T3E from *R. solanacearum* in yeast altered the TORC1 pathway, it was plausible that the effector had a similar effect in its natural context, i.e. when translocated inside the cells of plants infected by the pathogen. In plants, it has been shown that TOR silencing results in activation of nitrogen recycling activities and reduces primary nitrogen assimilation, measured by nitrate reductase activity^34^. In order to test whether *awr5* expression resulted in TOR inhibition in plants we thus used this activity as readout. Transient expression of *awr5* in *Nicotiana benthamiana* leaves resulted in a significant reduction of nitrate reductase activity compared to the control (GUS) (Fig. 7a). Leaky expression of *awr5* prior to induction may account for the slightly lower nitrate reductase activity values in leaves transformed with *awr*5. *awr5* expression did not significantly affect the activity of the TOR-independent, constitutive enzyme glucose-6-phosphate dehydrogenase (Fig. 7b). This clearly indicates that the decrease in the TOR- dependent nitrate reductase activity is specifically caused by *awr5* expression in plants.

The mechanisms by which AWR5 alters the TOR pathway in plants remains to be determined. Transient expression of *awr5* did not result in autophagy induction in *N. benthamiana* leaves expressing the autophagy marker GFP:ATG8a (Supplementary Fig. S11a). In addition, we could not detect direct interaction between AWR5 and TOR1 by co-immunoprecipitation using *N. benthamiana* leaves transiently over-expressing tagged versions of the two proteins (Supplementary Fig. S11b).

To further prove that AWR5 impacts the plant TOR pathway we infected *Arabidopsis thaliana* wild-type Col-0 plants, TOR1-silenced plants (*TOR* RNAi)^35^ and two mutant lines disrupted in the genes encoding either of the *CDC55* homologues (*b55α* and *b55β*)^36^ with *R. solanacearum* and recorded the appearance of wilting symptoms over time. TOR1-silenced lines were slightly more resistant to bacterial infection (Fig. 7c) and the two lines mutated in the *CDC55* homologues showed a striking resistance to infection as compared to the wild-type *Arabidopsis* (Fig. 7d), indicating that AWR5 effector may be targeting the TOR pathway in both plants and yeast. Although TOR RNAi lines have been previously reported to be slightly reduced in growth compared^35^, in our growing conditions both TOR RNAi and b55 mutants were indistinguishable from wild-type plants (Fig. S12), ruling out the possibility that their altered response to *R. solanacearum* infection was due to reduced surface of interaction.

## Discussion

In this work, we have produced *R. solanacearum* AWR effectors in yeast and have found that AWR5 impacts the TORC1 pathway, an essential component of eukaryotic cells. The premise for using *Saccharomyces cerevisiae* was that this organism carries out most eukaryotic processes and, unlike the host cells where T3E are naturally injected, it shows less gene redundancy and lacks resistance components that counteract and mask effector function^37^. For instance, gain-of-function analyses of T3E in plants are often hampered by a hypersensitive response (HR), a programmed cell death associated with recognition of effectors or effector virulence activities^38^.

A number of studies have successfully used *S. cerevisiae* as a model to identify T3E targets^8^,^13^. Toxicity -ranging from growth arrest to cell death- is the most common phenotype observed in these studies. However, this is not a widespread phenomenon when *R. solanacearum* T3E are expressed in yeast, as only 6 out of 36 effectors representing the repertoire of strain GMI1000 caused substantial growth inhibition (this work and^39^). Interestingly, four out of the six toxic T3E encode AWR proteins, suggesting a distinct function for this effector family in bacterial-host interactions. Cell growth inhibition caused by T3E has been traced back to interference on vesicle trafficking, disruption of the cytoskeleton or MAP Kinase alteration^8^, providing important clues on T3E function. In the case of AWR5, we show that it targets a novel cellular process, namely, the TORC1 pathway.

As mentioned above, the TORC1 protein complex regulates the transition between growth and quiescence in response to nutrient status and can be inhibited by rapamycin. TORC1 acts by controlling three major cell components: the kinase Sch9, Tap42, its associated phosphatases and the ATG1 complex^14^,^15^. Active TORC1 directly phosphorylates Sch9 -the orthologue of the mammalian S6 kinase-, which induces RiBi genes, such as *STM1* and *NSR1*, to increase translation and promote growth (Fig. 1a). In addition, when TORC1 is active, the essential downstream regulatory protein, Tap42, is phosphorylated and associates with the catalytic subunits of the PP2A and PP2A-like phosphatases, which are retained in membranes interacting with TORC1^14^. Finally, active TORC1 can inhibit autophagy by phosphorylation of ATG13, which prevents association with the ATG1 kinase and subsequent autophagy induction^32^. On the contrary, when TORC1 is inactivated by rapamycin treatment or nitrogen starvation, Tap42 and the PP2A and PP2A-like phosphatases are released to the cytosol and activated, allowing expression of stress genes and NCR genes such as *GAP1* and *MEP2*^15^ (Fig. 1a). This gene reprogramming takes place through PP2A-mediated inhibition of nuclear export of the Msn2/4 factors and PP2A/Sit4-mediated dephosphorylation and subsequent translocation of Gln3 to the nucleus. Our gene expression analyses and biochemical characterizations showed that the bacterial effector AWR5 interferes with the TORC1-regulated pathways, repressing ribosome biogenesis and translation and activating autophagy and stress responses. Activation of the latter, which are incompatible with growth, could explain the dramatic growth defects triggered by AWR5 in yeast. Our findings that mutants in two PP2A subunits (*cdc55* and *tpd3)* totally rescued this phenotype strongly support that AWR5 impacts TORC1-regulated pathways in eukaryotic cells.

As mentioned before, most TORC1-controlled effects occur through two major effector branches, mediated by the Sch9 kinase and by complexes of Tap42 and the phosphatases (mainly PP2A and Sit4). The wide transcriptomic impact of AWR5 on all TORC1-controlled pathways, mimicking the effect of rapamycin or nitrogen starvation, could be explained by assuming that AWR5 targets multiple hits downstream the pathway. Along this line, downstream components of the TOR pathway have already been involved in plant defense: PP2A was found to negatively regulate pathogen perception^40^ and PP1A is targeted by a *Phytophtora infestans* effector^41^,^42^. However, the most likely scenario is that AWR5 would target a few or even a single target controlling all these processes. If this were the case, AWR5 would exert its function inhibiting TORC1 upstream of PP2A, thus causing Sch9 inhibition, autophagy activation and the release of Tap42 and PP2A phosphatase subunits. The notion of a single target is reinforced by the fact that only a limited number of T3E molecules are injected into the host cell to exert their function. Along this line, leaky expression of *awr5* from a tet-off promoter in the presence of the repressor doxycycline had a detectable effect on yeast growth.

The observation that deletion of genes encoding two components of the PP2A heterotrimeric forms, *CDC55* and *TPD3*, abolishes the dramatic growth defect of cells expressing *awr5* suggests that in spite of the wide transcriptional effect caused by *awr5* expression, the major reason for AWR5 toxicity lies downstream PP2A and indicates that the formation of this heterotrimer is essential for the negative effect of AWR5 to take place. In this regard, it is worth noting that deletion of *TPD3* and of *CDC55* yields yeast cells resistant to rapamycin, whereas that of *RTS1* does not. Moreover, it has been proposed that an active TORC1 pathway promotes the association of Tap42 with PP2A catalytic subunits Pph21/22 to form complexes necessary for sustaining cell growth, whereas Cdc55 and Tpd3 would inhibit such association^43^. Although our study does not allow pointing to a specific TOR-regulated event to explain the inhibitory effect of AWR5, the observation that deletion of *CDC55* only normalizes the expression of specific subsets of genes altered by *awr5* expression (i.e. NCR genes but not ribosomal protein encoding genes) or the fact that AWR5-mediated autophagy promotion was not dependent on Cdc55 contribute to narrow the possible candidates.

Interestingly, during the course of this work, the *cdc55* mutant has been also isolated in a screen for suppressors of the yeast growth inhibition caused by the *Erwinia amylovora* T3E DspA^12^. This could suggest that the PP2A phosphatase has evolved as a cellular hub, targeted by different pathogens to interfere with plant host cell homeostasis. However, DspA caused a specific alteration of the yeast sphingolipid biosynthesis, showing no overlap with AWR5-triggered phenotypes other than the Cdc55-dependent growth inhibition. In addition, AWR5 still caused its toxicity on strains with mutations in the small GTPase *rho2* and in the sphingolipid biosynthesis gene *sur1* (data not shown), which strongly supressed DspA-triggered growth defects^12^. All these data support a different mode of action for these T3Es, only sharing Cdc55 as an intermediate in signal transduction.

TOR functions are conserved across kingdoms; in plants TOR is also a master regulator of the cell, controlling the switch between stress and growth^44^,^45^. Our data clearly supports the idea that AWR5 alters the TOR pathway in plants.

First, *awr5* expression *in planta* results in nitrate reductase activity inhibition. This enzyme has a central role in nitrogen metabolism and its inhibition has been previously linked to TOR deficiency and activated nitrogen recycling^34^. Noteworthy, even a minimal escape in *awr5* expression visibly impacted plant nitrate reductase activity, similar to the yeast growth inhibition caused by leaky expression of *awr5*. This strengthens the notion of a conserved AWR5 function as an extremely efficient modulator of the TOR pathway in disparate eukaryotic contexts. Second, TOR-deficient plants were more susceptible *to R. solanacearum* infection and plants lacking the *CDC55* homologues *B55α* or *B55β*, showed enhanced resistance to the pathogen. These opposite results are expected if the bacterium inhibits TOR signalling, as the B55 activity is repressed by TOR, and demonstrate a novel role for the TOR complex in plant defence.

In the context of *Ralstonia solanacearum* infection it remains a mystery why a bacterial T3E would mimic the effect of nitrogen starvation on infected tissues. Interestingly, there are several instances in the literature showing modulation of the host metabolism by T3Es. For example, the *R. solanacearum* effector RipTPS was shown to possess trehalose-6-phosphate synthase activity^46^ and the effector WtsE from *Pantoea stewartii* was shown to alter phenylpropanoid metabolism^47^.

Furthermore, group A *Streptococcus* enhances its growth by activation of asparagine metabolism via ER stress induction in mammalian cells^48^. Since ER stress responses are intimately connected with TOR signalling^49^, it is tempting to speculate that AWR5 modulates the TOR pathway to induce ER stress responses and stimulate growth by an analogous mechanism to the one proposed in *Streptococcus*. On an alternative hypothetic scenario, AWR5-mediated inhibition of TOR (nitrogen recycling, autophagy, inhibition of protein synthesis…) would be beneficial for the bacterium during the last stages of infection, as it would facilitate plant cell dismissal and consequently nutrient availability.

## Methods

### Plasmids, strains and gene cloning

All strains and plasmids used in this study are described in Supplementary Table S1. For heterologous expression of *awr*s under the control of the galactose inducible promoter (*GAL1*), expression vectors were constructed by recombining entry clones carrying each of the *awr* ORFs into the Gateway destination vector pAG426GAL-ccdb-HA^50^ through a Gateway LR reaction (Invitrogen, Waltham, Massachusetts, USA). For expression of *awr5* fragments in yeast, N-terminal (1368 bp) and C-terminal (1821 bp) halves of *awr5* as well as a central (1425 bp) fragment overlapping them were amplified from genomic DNA.

For integration of the *awr* genes fused to a C-terminal GFP tag at the locus of *URA3* gene in the yeast chromosome, each of them was cloned by Gateway recombination or ligation into the integrative vector pYI-GWY, a *URA3* plasmid in which the heterologous genes are under the control of a Tet-off promoter created in this study. Following linearization with *Bst*BI that cuts inside in *URA3* cassette, pYI-GWY derivatives carrying genes *awr1* to *awr5* were integrated into the yeast chromosome by double recombination into the *URA3* locus in yeast. To this end, the wild type strain JA-100 containing a *ura3* point mutation was used as recipient, giving rise to uracyl autotrophs after *awr* integration. For expression of *awr5* gene in the *cdc55*Δ mutant yeast strain, cloning was performed in two steps. Firstly, a *cdc55::KanMX4* cassette from the *cdc55*Δ strain in the BY4741 background was amplified and subsequently introduced into the genome of strain JA-100. Secondly, the *awr5* gene fused to the C-terminal GFP was integrated into the newly constructed *cdc55* strain as described above.

### Yeast strains and growth conditions

For expression of *awrs* or their fragments under the control of the galactose promoter, yeast cells were grown for 2 days in SD-Ura + raffinose 2%, then diluted to optical density at 600 nm of 0.4 in water and plated either in repressing media (glucose) or inducing media (galactose) to monitor the effects of AWRs in cell growth/viability. For standard growth inhibition experiments on plates, strains were incubated overnight with shaking in selective medium with doxycycline 20 μg/ml. Cultures were then normalized to OD_600_ = 0.1–0.2 and grown until exponential phase. 1 OD_600_ of cells were then harvested, washed 2 times with sterile water, re-suspended in 1 ml water and 10-fold serially diluted in water four times. Each suspension (5 or 10 μl) was dropped either in non-inducing media (+doxycycline) or inducing media (no doxycycline) onto agar plates and then incubated for 2–3 days before photographs were taken.

To test growth viability in liquid media over time and for sample harvesting for RNA isolation, yeast strains were grown overnight in rich YPD medium with doxycycline 15 μg/ml (repressing conditions), then normalized to OD_600_ = 0.05 and grown for 2, 4, 6 or 8 hours in YPD+dox (non-inducing conditions) and YPD (inducing conditions). Similar growth conditions were carried out for protein extraction and beta-galactosidase assays, using selective medium in this case. To test viability of yeast cells expressing *awr5* after doxycycline addition, strains were grown overnight in either SD-Ura+dox (non-inducing conditions) or SD-Ura (inducing). Cells were recovered and normalized to OD_600_ = 0.05 and grown in liquid in SD-Ura+dox. Samples were harvested at different time points, serially 10-fold diluted and plated onto solid SD-Ura+dox and incubated for 2 days at 28 °C until colonies were counted.

For methylene blue staining, yeast cells carrying *awr5* were harvested at 6 hours after induction and stained for 5 minutes with a 0.01% methylene blue solution in glycine buffer. In parallel, the same cells were fixed with formaldehyde 37% for 10 mins before methylene blue addition as a positive staining control. Images were obtained using a Dapi 395–440/FT 460/LP470 filterset.

To measure yeast cell size, wild-type yeast strains (JA-100) and strains bearing *awr5* were grown overnight in YPD medium with and without doxycycline (15 μg/ml). The next day, cultures were normalized to OD_600_ = 0.05 and grown in liquid either in YPD+dox or YPD during 6 and 8 hours. Cells were analyzed with a Scepter Handheld Automated Cell Counter (Merck Millipore, Darmstadt, Germany).

To measure induction of autophagy, wild-type and *cdc55*Δ strains carrying *awr5* and ATG8-GFP were grown overnight in selective media plus doxycycline. Cultures were then normalized to an OD_600_ = 0.2, grown until exponential phase, normalized again to OD_600_ = 0.05 and finally grown overnight with or without doxycycline until samples were harvested. For autophagy induction after nitrogen starvation JA-100 cells were grown overnight in SD medium without ammonium sulfate (BD Difco, Franklin Lakes, NJ, USA) and 2% glucose.

### DNA microarray analysis

Aliquots of the same samples harvested to test viability of cells expressing *awr5* in liquid media at 2, 4 and 6 hours after induction were used for microarray analysis. For microarray hybridization, total RNA (8 *μ*g) was employed for cDNA synthesis and labelling using the indirect labelling kit (CyScribe Post-Labeling kit; GE Healthcare, Wauwatosa, WI, USA) with Cy3–dUTP and Cy5–dUTP fluorescent nucleotides. The cDNA obtained was dried, re-suspended in hybridization buffer and evaluated with a Nanodrop spectrophotometer (Nanodrop Technologies, Thermo Scientific, Waltham, MA, USA). The combined fluorescently labelled cDNAs were hybridized to yeast genomic microchips constructed in our laboratory by arraying 6014 different PCR-amplified open reading frames from *S. cerevisiae*^51^. Microarrays were processed as described previously^52^, scanned with a ScanArray 4000 apparatus (Packard BioChip Technologies, Perkin Elmer, Waltham, MA, USA) and the output was analysed using GenePix Pro 6.0 software. Data collected from 2 biological replicates (two microarrays each, with dye swap) after 2, 4 and 6 h of doxycycline removal (thus triggering expression of *awr5*) were combined. Genes were considered induced or repressed by AWR5 expression when the minus/plus doxycycline ratio was ≥2.0 or ≤0.5, respectively, for both biological replicates. All data has been added to the Gene Expression Omnibus (GEO) database under numbers GSE70202, GSE70331 and GSE70835.

### qRT-PCR

Two independent biological replicas of the strain carrying *awr5* grown in inducing and non-inducing conditions were harvested at 4 and 6 hours after induction and subjected to RNA extraction to quantify *awr5* mRNA levels, whereas of *GAP1, MEP2, STM1* and *NSR1* levels were only tested from samples obtained 6 h after induction. For quantitative real-time PCR, a Light Cycler 480 (Roche, Basel, Switzerland) with SYBR Green chemistry was used with three technical replicas. Actin was used as a housekeeping gene to normalize samples.

### RNA-seq experiments

Libraries were prepared with the QuantSeq 3′ mRNA kit (Lexogen, Greenland, NH, USA) using 0.5 μg of total RNA purified as above. Sequencing was performed in an Illumina MiSeq machine with Reagent Kit v3 (single end, 80–125 nt/read). Two biological replicates were sequenced, obtaining a total number of 8.4–12.9 million reads per condition. Mapping of fastq files to generate SAM files was carried out with the Bowtie2 software^53^ in local mode (95.1–97.3% mapped reads). The SAM files were analyzed with the SeqMonk software (www.bioinformatics.bbsrc.ac.uk/projects/seqmonk). Mapped reads were counted using CDS probes (extended 100 nt downstream the open reading frame because the library is biased towards the 3′-end of mRNAs) and corrected for the largest dataset. Raw data was subjected to diverse filters to remove sequences with a low number of reads.

### Protein assays

For immunoblots, 30 or 40 OD_600_ units from overnight yeast cultures grown in non-inducing or inducing conditions were were resuspended in 500 μl of extraction buffer (50 mM Tris-HCl pH7.5, 1 mM EDTA, 0.1% Nonidet P-40, 1% glycerol, with complete protease inhibitor (Roche, Basel, Switzerland) and subjected to 10 cycles of 1 minute sonication and 1 minute pauses. Supernatants were recovered after centrifugation at 500 g for 10 min at 4 °C. 125 μg of total protein extracts were separated on polyacrylamide gels and immunoblot was performed using anti-GFP mouse monoclonal antibody (clone B-2; Santa Cruz Biotechnology, Dallas, TX, USA).

Beta-galactosidase activity was measured from, 2 ml of cultures pelleted 6 hours after induction as described^54^.

### Plant material

Wild type (Wt) Columbia 0, TOR-silenced 35-7 (*TOR* RNAi)^35^, *b55α* and *b55β Arabidopsis* mutant lines^36^ were used. 3 to 4-week-old *N. benthamiana* plants were used for transient expression experiments.

### Enzymatic activity determinations

To measure nitrate reductase activity, *N. benthamiana* plants were treated two times a week with 2mM-15mM KNO_3_, then, transient *Agrobacterium*-mediated transformation was performed as previously described^20^ using the estradiol-inducible vector pMDC7 carrying AWR5 or GUS. Protein expression was induced by painting the leaves 14 hours post-agrobacterium infiltration with 20 μM estradiol and Silwet L-77 adjuvant. Whole leaves (1 g) were harvested at 0 and 1 hour after post-estradiol induction and homogenized in 3 ml of 0.1 M HEPES-KOH, pH 7.5, 3% polyvinylpolypyrrolidone, 1 mM EDTA and 10 mM cysteine. The extracts were filtered through four layers of Miracloth (Merk Millipore, Billerica, USA) and centrifuged for 15 minutes at 30,000 × g at 4 °C and nitrate reductase activity measured as described in^55^.

To measure glucose-6-phosphate dehydrogenase activity *N. benthamiana* leaves were transiently transformed as previously described^20^ using the estradiol-inducible vector pMDC7 carrying AWR5 or GUS. Protein expression was induced by painting the leaves with 20 μM estradiol and Silwet L-77 adjuvant 14 hours post-agrobacterium infiltration. Half-leaves (500 mg) were harvested at 0 and 1 hour after post-estradiol induction and homogenized in 500 μl of 20 mM imidazol, pH 7. The extracts were centrifuged 15 minutes at 1000 × *g* at 4 °C and the supernatant was transferred to a new tube and kept on ice. To determine the activity of glucose-6-phosphate dehydrogenase activity 170 μl of 2x assay buffer (0.1 M imidazol, 0.2 M KCl, 20 mM MgCl2, 2 mM EDTA), 131 μl H_2_O, 7 μl of 10 mM NADP and 25 μl of cell-free extract were sequentially added to a spectrophotometer cuvette and the A_340_ was monitored for a few minutes until stabilization. Then 7 μl of 50 mM glucose-6-phosphate were added and the A_340_ was recorded, as a measure of Glucose-6-phosphate dehydrogenase activity (expressed as nmoles min^−1^ mg^−1^ protein).

### Pathogenicity assays

*R. solanacearum* pathogenicity tests were carried out using the soil-drench method as described^56^.

## Additional Information

**How to cite this article**: Popa, C. *et al.* The effector AWR5 from the plant pathogen *Ralstonia solanacearum* is an inhibitor of the TOR signalling pathway. *Sci. Rep.* **6**, 27058; doi: 10.1038/srep27058 (2016).

## Supplementary Material

Supplementary Information
