# Author Correction: The effector AWR5 from the plant pathogen *Ralstonia solanacearum* is an inhibitor of the TOR signalling pathway

**DOI:** 10.1038/s41598-020-78190-9

**Published:** 2020-11-26

**Authors:** Crina Popa, Liang Li, Sergio Gil, Laura Tatjer, Keisuke Hashii, Mitsuaki Tabuchi, Núria S. Coll, Joaquín Ariño, Marc Valls

**Affiliations:** 1 Centre for Research in Agricultural Genomics (CSIC-IRTA-UAB-UB), Bellaterra, Catalonia Spain; 2 Genetics Department, Universitat de Barcelona, Barcelona, Catalonia Spain; 3 Institut de Biotecnologia i Biomedicina and Departament de Bioquímica i Biologia Molecular, Universitat Autònoma de Barcelona, Cerdanyola del Vallès, Catalonia Spain; 4 Laboratory of Applied Molecular and Cell Biology, Kagawa University, Kagawa, Japan

Correction to: *Scientific Reports* https://doi.org/10.1038/srep27058, published online 03 June 2016

An article contains an error on page 8 of the article:

“TOR1-silenced lines were slightly more resistant to bacterial infection (Fig. 7c) and the two lines mutated in the CDC55 homologues showed a striking resistance to infection as compared to the wild-type Arabidopsis (Fig. 7d), indicating that AWR5 effector may be targeting the TOR pathway in both plants and yeast.”

Should read:

“TOR1-silenced lines were slightly more susceptible to bacterial infection (Fig. 7c) and the two lines mutated in the CDC55 homologues showed a striking resistance to infection as compared to the wild-type Arabidopsis (Fig. 7d), indicating that AWR5 effector may be targeting the TOR pathway in both plants and yeast.”

