# Supplementary material for: The effector AWR5 from the plant pathogen Ralstonia solanacearum is an inhibitor of the TOR signalling pathway
Source: Sci Rep. 2016 Jun 3;6:27058. doi: 10.1038/srep27058 (PMC4891724; doi:10.1038/srep27058)
Supplement: Supplementary Information [file srep27058-s1.pdf]

The effector AWR5 from the plant pathogen *Ralstonia solanacearum* is an inhibitor of the TOR signalling pathway

Crina Popa[1,2], Liang Li[1], Sergio Gil[2], Laura Tatjer[3], Keisuke Hashii[4], Mitsuaki Tabuchi[4], Núria S. Coll[1]*, Joaquín Ariño[3] and Marc Valls[1,2]

[1] Centre for Research in Agricultural Genomics (CSIC-IRTA-UAB-UB), Bellaterra, Catalonia, Spain

[2] Genetics Department, Universitat de Barcelona, Barcelona, Catalonia, Spain

[3] Institut de Biotecnologia i Biomedicina and Departament de Bioquímica i Biologia Molecular, Universitat Autònoma de Barcelona, Cerdanyola del Vallès, Catalonia, Spain

[4] Laboratory of Applied Molecular and Cell Biology, Kagawa University, Kagawa, Japan

* Address correspondence to: Nuria S. Coll

Centre for Research in Agricultural Genomics (CSIC-IRTA-UAB-UB).

Campus UAB. Edifici CRAG. Bellaterra, 08193. Catalonia, Spain

E-mail: nuria.sanchez-coll@cragenomica.es

Supplementary Materials

Co-immunoprecipitation assays

Yeast co-immunoprecipitations were performed as follows: overnight yeast cultures grown in inducing conditions were diluted to an OD_{600} of 0.05 and resuspended in 500 µl of extraction buffer (50 mM Tris-HCl pH7.4, 100 mM NaCl, 1% Triton X-100) supplemented with 2 mM DTT, 1 mM PMSF and complete protease inhibitor (Roche, Basel, Switzerland). 300 µl of Zirconia glass beads (BioSpec Products, Bartlesville, USA) were added and the suspension homogenized using a FastPrep (MP Biomedicals, Santa Ana, USA) for 5 cycles of 45 seconds each. Samples were then centrifuged 10 minutes at 500 *x g* at 4ºC to remove cell debris and the resulting protein extract was diluted to 1.5 mg/ml. For AWR5-Cdc55 co-immunoprecipitation 1 ml of protein extract was incubated with 50 µl of magnetic GFP beads (MACS Miltenyi Biotec, Bergisch Gladbach, Germany) according to [1]. For Lst8-TAP 1 ml of protein extract was incubated with 60 µl of rabbit IgG agarose beads (Sigma-Aldrich, Buchs, Switzerland) for 2 h at 4ºC under constant rotation. The beads were sequentially washed: 1 time with extraction buffer, 3 times with extraction buffer supplemented with 500 mM NaCl and 2 times with a buffer containing 50 mM Tris pH 8 and 2 mM DTT. Bead elution was performed by adding 30 µl of SDS 5x loading buffer and incubating 5 minutes at 95ºC. The samples were then centrifuged 1 minute at 1200 *x g* and 30 µl of supernatant, containing the protein eluate, transferred to a new tube.

Plant co-immunoprecipitations were performed as in [1]. Briefly, leaves of 4-week-old *N. benthamiana* leaves were infiltrated with *Agrobacterium tumefaciens* GV3101 expressing the anti-silencing vector *p19* [2] together with

myc-TOR1 [3] alone or in combination with *Estradiol::AWR5-HA-citrine. awr5* expression was induced 24 hours post-infiltration by treating the leaves with 20 µM estradiol. Eight hours later 3 leaf disks per sample were snap-frozen in liquid nitrogen and 200 µl of ice-cold extraction buffer (10 mM Tris-HCl pH 7.5, 150 mM NaCl, 0.5 mM EDTA, 0.5% NP-40) supplemented with 1 mM PMSF and complete protease inhibitor (Roche, Basel, Switzerland) was added. Samples were ground on ice using a tissue grinder and centrifuged at 16000 *x g*, 10 minutes at 4ºC. The supernatant was diluted to 1.5 mg/ml and processed as described above for AWR5-Cdc55 co-immunoprecipitations.

For western blot analyses, 50 µl of input (total protein extract) and half of the total eluate volume were loaded per SDS-PAGE gel. Immunoblots were performed using anti-GFP mouse monoclonal antibody diluted 1:5000 (clone B-2, Santa Cruz Biotechnology, Dallas, TX, USA), monoclonal anti-HA-HRP (clone 3F10, Roche, Basel, Switzerland), monoclonal anti-myc diluted 1:1000 (clone E10, Santa Cruz Biotechnology, Dallas, TX, USA) or polyclonal anti-PAP diluted 1:8000 (Sigma-Aldrich, Buchs, Switzerland).

Yeast growth assay

Yeast wild-type or TORC2 temperature sensitive mutant, *tor2*^{ts} (pRS415-*tor2-21*/*tor2*Δ, gift from Dr. Scott D. Emr) [4]. cells carrying pRS425-P_{tetoff}-AWR5 plasmid and constitutive active or kinase dead mutant of Ypk2 expression plasmid, yEP352-*YPK2*^{D239A} or YPK2$^{D239A,\ K379A}$, respectively (gift from Dr. Yoshiaki Kamada) [5] were spotted on SD (-Ura, -Leu) with or without doxycycline (20 µg/ml) and incubated for 3 days at 26˚C or 35˚C. Suppression of TORC2 pathway by constitutive active Ypk2 was evaluated by its growth.

Actin staining

For localization of actin, cells were grown to early log phase, shifted to the appropriate temperature for 2 hours, fiexd in 3.7% formaldehyde, and stained with rhodamine-phalloidin (Molecular Probes) as described previously [6]. The images were obtained by fluorescent microscope using a rhodamine filter.

Supplementary Table 1: Plasmids and strains used in this study.

Name	Description	Background/ Source
**Plasmids for expression of AWRs genes and AWR5 fragments under control of galactose promoter**		
pAG426GAL-ccdb-HA	Renamed pGAL; 2 µ pGAL1-GWY-HA URA3 CmR AmpR	7
pDONR207	Gateway™ entry vector, CmR GmR	Invitrogen
pDONR-Nterm *awr5*	pDONR207 containing N-terminal fragment of AWR5 from *R. solanacearum* GMI1000 GmR	This study
pDONR-Cen *awr5*	pDONR207 containing central fragment of AWR5 from *R. solanacearum* GMI1000 GmR	This study
pDONR-Cterm *awr5*	pDONR207 containing C-terminal fragment of AWR5 from *R. solanacearum* GMI1000 GmR	This study
pGAL-Nterm *awr5*-HA	pGAL bearing N-terminal fragment of AWR5 from R. solanacearum GMI1000 URA3 AmpR	This study
pGAL-Cen *awr5*-HA	pGAL bearing central fragment of AWR5 from R. solanacearum GMI1000 URA3 AmpR	This study
pGAL-Cterm *awr5*-HA	pGAL bearing C-terminal fragment of AWR5 from R. solanacearum GMI1000 URA3 AmpR	This study
pDONR-*awr5*	pDONR207 containing AWR5 from *R. solanacearum* GMI1000 GmR	8
pENTR-*awr4*	pENTR/SD/D-Topo containing AWR4 from R. solanacearum GMI1000 KmR	9
pDONR-*awr3*	pDONR207 containing AWR3 from *R. solanacearum* GMI1000 GmR	8
pENTR-*awr2*	pENTR/SD/D-Topo containing AWR2 from R. solanacearum GMI1000 KmR	9
pDONR-*awr1*	pDONR207 containing AWR1 from *R. solanacearum* GMI1000 GmR	8
pDONR-*gfp*	pDONR201 containing *green fluorescent* control gene KmR	F. Monteiro et al unpublished
pGAL-*awr5*	pGAL destination vector expressing AWR5 from R. solanacearum GMI1000 URA3 AmpR	This study
pGAL-*awr4*	pGAL destination vector expressing AWR4 from R. solanacearum GMI1000 URA3 AmpR	This study
pGAL-*awr3*	pGAL destination vector expressing AWR3 from R. solanacearum GMI1000 URA3 AmpR	This study
pGAL-*awr2*	pGAL destination vector expressing AWR2 from R. solanacearum GMI1000 URA3 AmpR	This study
pGAL-*awr1*	pGAL destination vector expressing AWR1 from R. solanacearum GMI1000 URA3 AmpR	This study
pGAL-*gfp*	pGAL destination vector expressing *gfp* control gene URA3 AmpR	This study
**Plasmids for integration of AWRs into yeast genome**		
YIplac211	Yeast integrative vector URA3 AmpR	10
pMT735	CEN pTet-off GFP tag URA3 CmR AmpR	11
pDONR-*awr5*	pDONR207 containing AWR5 (no stop) from *R. solanacearum* GMI1000 GmR	8
pMT735-*awr5*	pMT735 destination vector expressing AWR5 from R. solanacearum GMI1000 URA3 AmpR	This study
pMT735-*awr1*	pMT735 destination vector expressing AWR1 from R. solanacearum GMI1000 URA3 AmpR	This study
pMT735-*awr3*	pMT735 destination vector expressing AWR3 from R. solanacearum GMI1000 URA3 AmpR	11
pYI-GWY	Kpn I-Sac I fragment from pMT735 cloned into YIplac211 same sites URA3 CmR AmpR	This study
pYI-*awr1*	Kpn I-Sac I fragment from pMT735-*awr1* cloned into YIplac211 same sites URA3 AmpR	This study
pYI-*awr3*	Kpn I-Sac I fragment from pMT735-*awr3* cloned into YIplac211 same sites URA3 AmpR	This study

pYI-*awr5*	Kpn I-Sac I fragment from pMT735-*awr5* cloned into YIplac211 same sites URA3 AmpR	This study
pYI-*awr2*	pYI-GWY destination vector expressing AWR2 from R. solanacearum GMI1000 URA3 AmpR	This study
pYI-*awr4*	pYI-GWY destination vector expressing AWR4 from R. solanacearum GMI1000 URA3 AmpR	This study
**Plasmids for autophagy and β galactosidase assays**		
pRS415-ATG8-GFP	pRS415 ATG8-GFP *CEN LEU2* AmpR	[12]
YEp367R	Yeast shuttle vector for construction of *lacZ* fusions (*LEU2*, AmpR)	[13]
YEp357-*pGAP1*	YEp357 carrying PromGAP1(-968 to +21)::lacZ cloned Kpn I-XbaI URA3 AmpR	[14]
YEp367R-*pGAP1*	YEp367R carrying a fusion between lacZ gene and *GAP1* promoter LEU2 AmpR	This study
YCplac111-*pMEP2*	Yeast shuttle vector YCplac111 carrying Prom*MEP2*(-828 to +27)::lacZ LEU2 AmpR	[15]
**Plasmids for growth assays of TOR-related mutants**		
pMT921	pMT735 inverse PCR self-ligated plasmid; CEN pTet-off GFP tag URA3 AmpR	[11]
pMT830	2µ pTet-off, GFP tag URA3 CmR AmpR	[11]
pMT830-*awr5*	pMT830 destination vector expressing AWR5 from R. solanacearum GMI1000 URA3 AmpR	This study
YEplac112	2µ TRP1 AmpR	[10]
YEplac112-*awr5*	2µ YEplac112 Tet-Off promoter::AWR5::GFP TRP1 AmpR	This study
**Plasmids for overexpression of TOR-related genes**		
YEplac181-PPH21	2µ plasmid for *PPH21* gene expression under its own promoter (*LEU2*, AmpR)	[16]
YEplac181-PPH22	2µ plasmid for *PPH22* gene expression under its own promoter (*LEU2*, AmpR)	[16]
YEplac181-SIT4	2µ plasmid for *SIT4* gene expression under its own promoter (*LEU2*, AmpR)	[16]
YEp351-HAL3	2µ plasmid for *HAL3* gene expression under its own promoter (*LEU2*, AmpR)	[17]
**Plasmids for overexpression of TOR-related genes**		
pMDC7-*citrine*	Gateway destination vector, estradiol-inducible, Ct-YFPv/HA tag CmR SpR	E. Washington et al., unpublished
pMDC7-*awr5*	pMDC7 containing AWR5 from *R. solanacearum* GMI1000 SpR	[8]
pMDC7-*gus*	pMDC7 containing the *beta-glucuronidase* control gene SpR	[8]
pEDV6	Gateway destination vector for effector gene fusion to AvrRPS4 N-terminus (1-137). RPS4 promoter, HA tag RifR GmR	[18]
pEDV6-*awr*5	pEDV6 vector carrying *R. solanacearum* GMI1000 AWR5, GmR	[8]
**Yeast strains**		
BY4741	*MATa his3Δ0 leu2Δ0 met15Δ0 ura3Δ0*	[19]
JA-100	*MATa ura3-52 leu2-3,112 trp1-1 his4 can-1*^{r}	[20]
JA-100-GFP	JA-100 *GFP::URA3*	This study
JA-*awr5*	JA-100 *AWR5::GFP::URA3*	This study
JA-*awr4*	JA-100 *AWR4::GFP::URA3*	This study
JA-*awr3*	JA-100 *AWR3::GFP::URA3*	This study
JA-*awr2*	JA-100 *AWR2::GFP::URA3*	This study
JA-*awr1*	JA-100 *AWR1::GFP::URA3*	This study
gln3Δ	BY4741 *gln3Δ::kanMX4*	EUROSCARF
tip41Δ	BY4741 *tip41Δ::kanMX4*	,EUROSCARF
ppm1Δ	BY4741 *ppm1Δ::kanMX4*	EUROSCARF

cdc55Δ	BY4741 *cdc55Δ::kanMX4*	EUROSCARF
rts1Δ	BY4741 *rts1Δ::kanMX4*	EUROSCARF
tpd3Δ	BY4741 *tpd3Δ::kanMX4*	EUROSCARF
W303-1A	*MAT*a *leu2-3,112 trp1-1 can1-100 ura3-1 ade2-1 his3-11,15*	[21]
DEY172-2B	*W303-1A pph22-172::URA3 pph21A1::HIS3*	[22]
cdc55Δ	JA-100 *MATa cdc55Δ::kanMX4*	JA-100, This study
cdc55Δ AWR5-GFP	JA-100 *MATa cdc55Δ::kanMX4 AWR5::GFP::URA3*	JA-100, This study

***Agrobacterium tumefaciens* strains**

GV3101 *awr5*	GV3101 expressing AWR5-YFP-HA RifR GmR SpR	GV3101, This study
GV3101 *gus*	GV3101 expressing GUS-YFP-HA RifR GmR SpR	GV3101, This study

***Pseudomonas syringae* strains**

DC3000	*P.syringae* pv. tomato DC3000 strain, RifR	[23]
3000-*awr5*	DC3000 expressing RPS4N-HA-AWR5, RifR GmR	[8]

***E. coli* strains**

MACH 1	*ΔrecA1398 endA1 tonAΦ80ΔlacM15 ΔlacX74 hsdR*(r_K^- m_K^+)	Invitrogen
DB3.1	*F- gyrA462 endA1 glnV44 Δ(sr1-recA) mcrBmrr hsdS20*(r_B^-, m_B^-) *ara14 galK2 lacY1 proA2 rpsL20(Smr) xyl5 Δleu mtl1, Smr*	Invitrogen

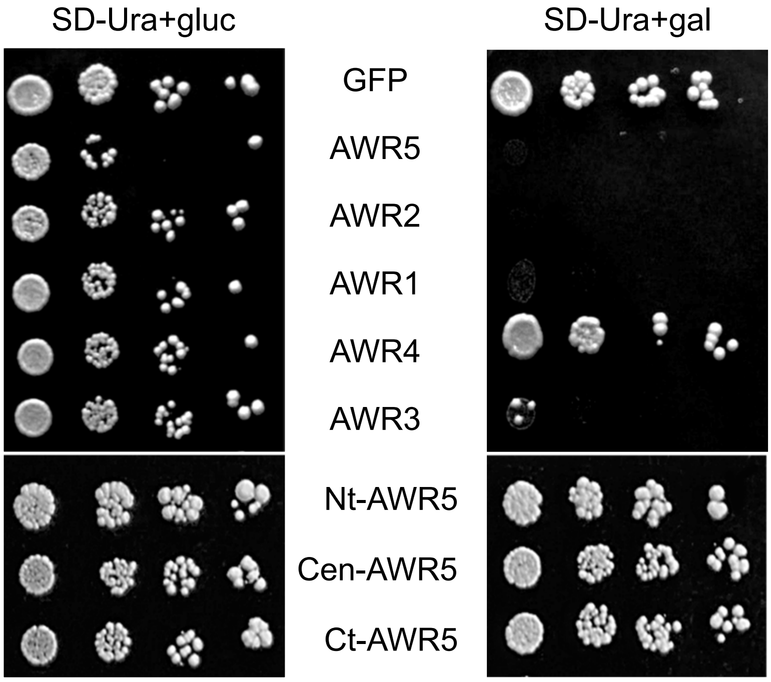

Supplementary Figure S1. Full-length AWR5 causes growth inhibition in yeast when expressed from a high-copy-number plasmid. Yeast strains carrying the awr5 effector gene or its N-terminal (Nt-AWR5), central (Cen-AWR5) and C-terminal (Ct-AWR5) fragments or a control (GFP) gene were subjected to serial 10-fold dilutions and spotted onto solid SD-Ura+gluc (glucose - repressing medium) and SD-Ura+gal (galactose - inducing medium). Photographs were taken after 2 days of growth.

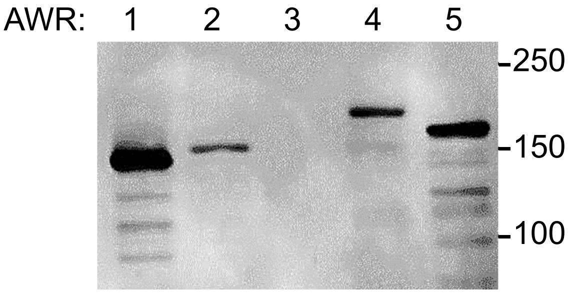

Supplementary Figure S2. Protein levels of the different AWR family members expressed in yeast. Total protein was extracted from yeast strains expressing AWR effectors fused to GFP and immunoblotted using anti-GFP antibody.

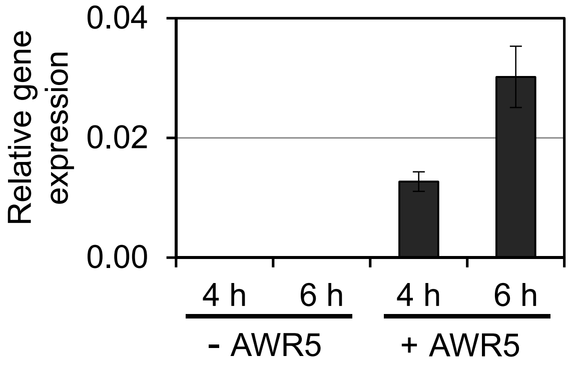

Supplementary Figure S3. qRT-PCR showing *awr5* expression levels relative to actin. Yeast strains bearing *awr5* gene were grown in SD-Ura+dox (-AWR5) and SD-Ura (+AWR5). *awr5* gene expression was tested at 4 and 6 hours after induction. Error bars represent standard errors of 2 independent clones.

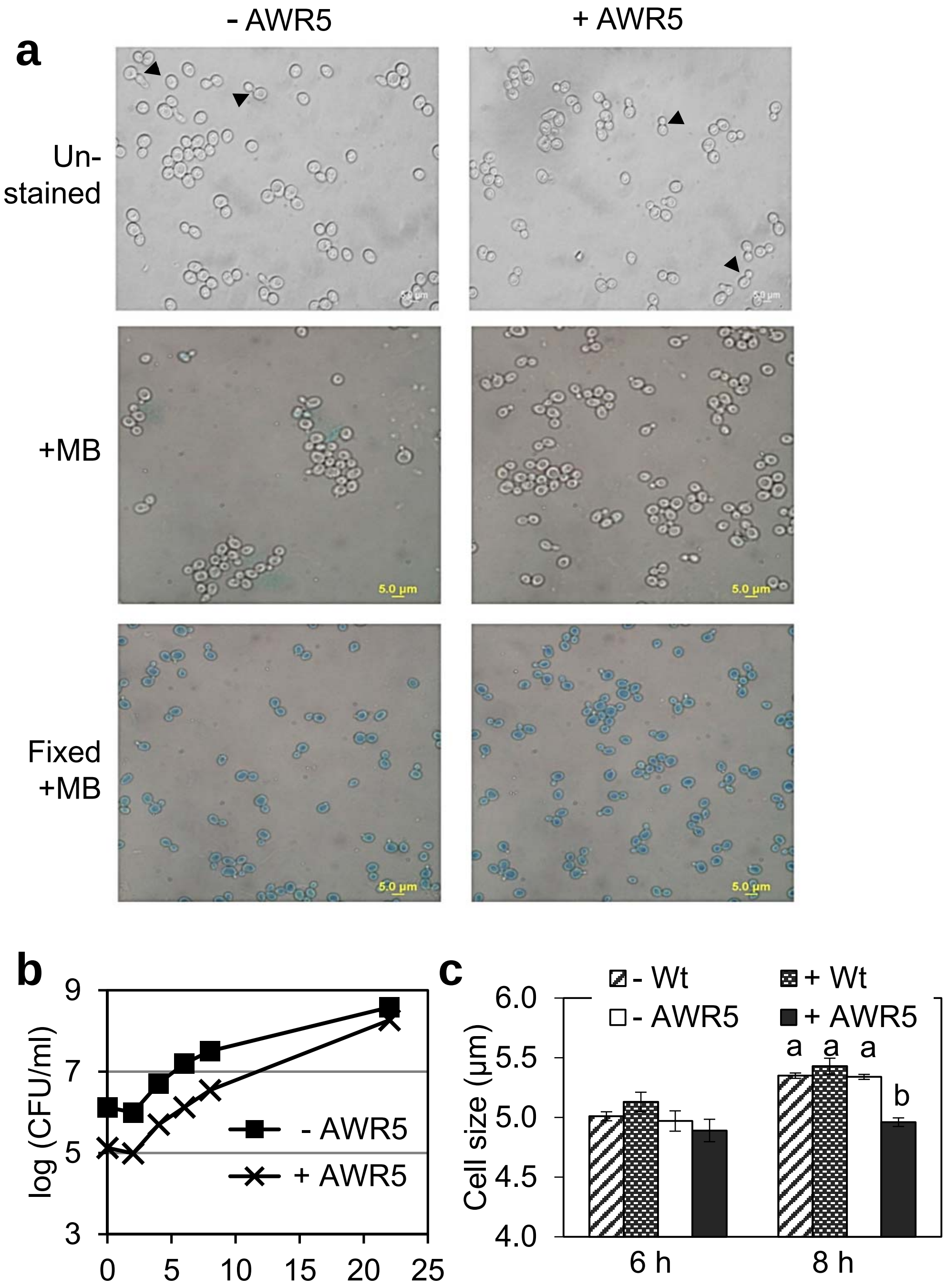

Supplementary Figure S4. *awr5* does not cause cell cycle arrest nor cell death in yeast. (A) Representative bright field microscope pictures of cells bearing *awr5* grown for 6 hours in SD-Ura+dox (-AWR5) in SD-Ura (+AWR5). Upper panel: unstained cells. Middle panel: Cells stained with methylene blue. Lower panel: Cells fixed with formaldehyde before methylene blue staining. Black arrows indicate budding yeast cells. Bars correspond to 5 µm. (B) Growth curves under repressing conditions (SD-Ura+dox) of the yeast strain harboring *awr5* that had been previously been grown in SD-Ura+dox (repression of *awr5*) or SD-Ura (induction of *awr5*). Yeast counts were measured by spotting culture dilutions on SD-Ura+dox plates. The logarithm of colony forming units (CFU) per ml is shown over time. A representative curve is shown for each condition. (C) Expression of AWR5 diminishes yeast cell size. Strains bearing AWR5 were grown in YPD+dox (repression, -AWR5) or YPD (induction, +AWR5) for 6 and 8 hours. Cell size was analyzed with a Scepter Handheld Automated Cell Counter (Merck Millipore) and compared to that of the wildtype strain with (-Wt) and without doxycycline (+Wt). Letters at 8 hpi indicate a statistically significant difference following post-ANOVA Tuckey test (*P*<0.001).

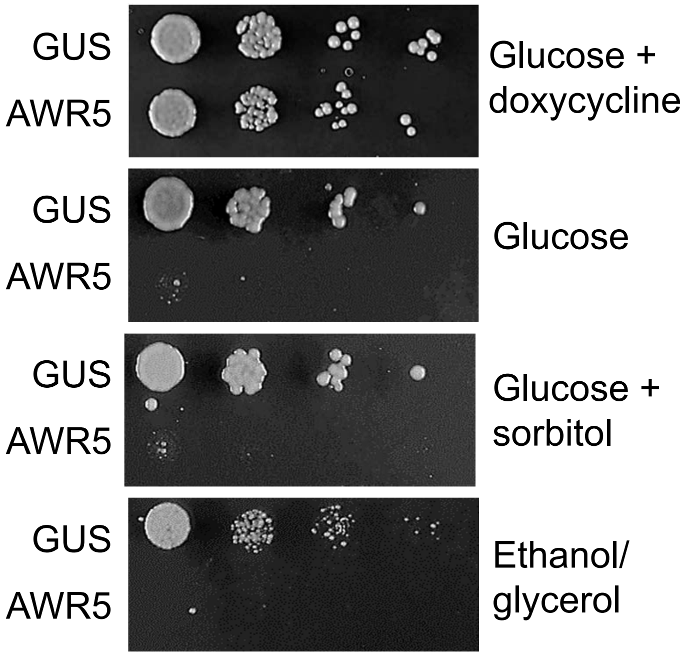

Supplementary Figure S5. AWR5-mediated yeast growth inhibition is not rescued by osmoprotection or respiration. Serial tenfold dilutions of strains bearing *awr5* or a control gene (*GUS)* were spotted onto solid SD-Ura medium containing glucose and doxycycline (repressing conditions), or on plates without doxycyline (*awr5* inducing conditions) supplemented either with: glucose, glucose and sorbitol (osmoprotectant) or ethanol/glycerol (carbon sources that force respiration). Photographs of representative plates out of several replicas were taken after 2 days of growth.

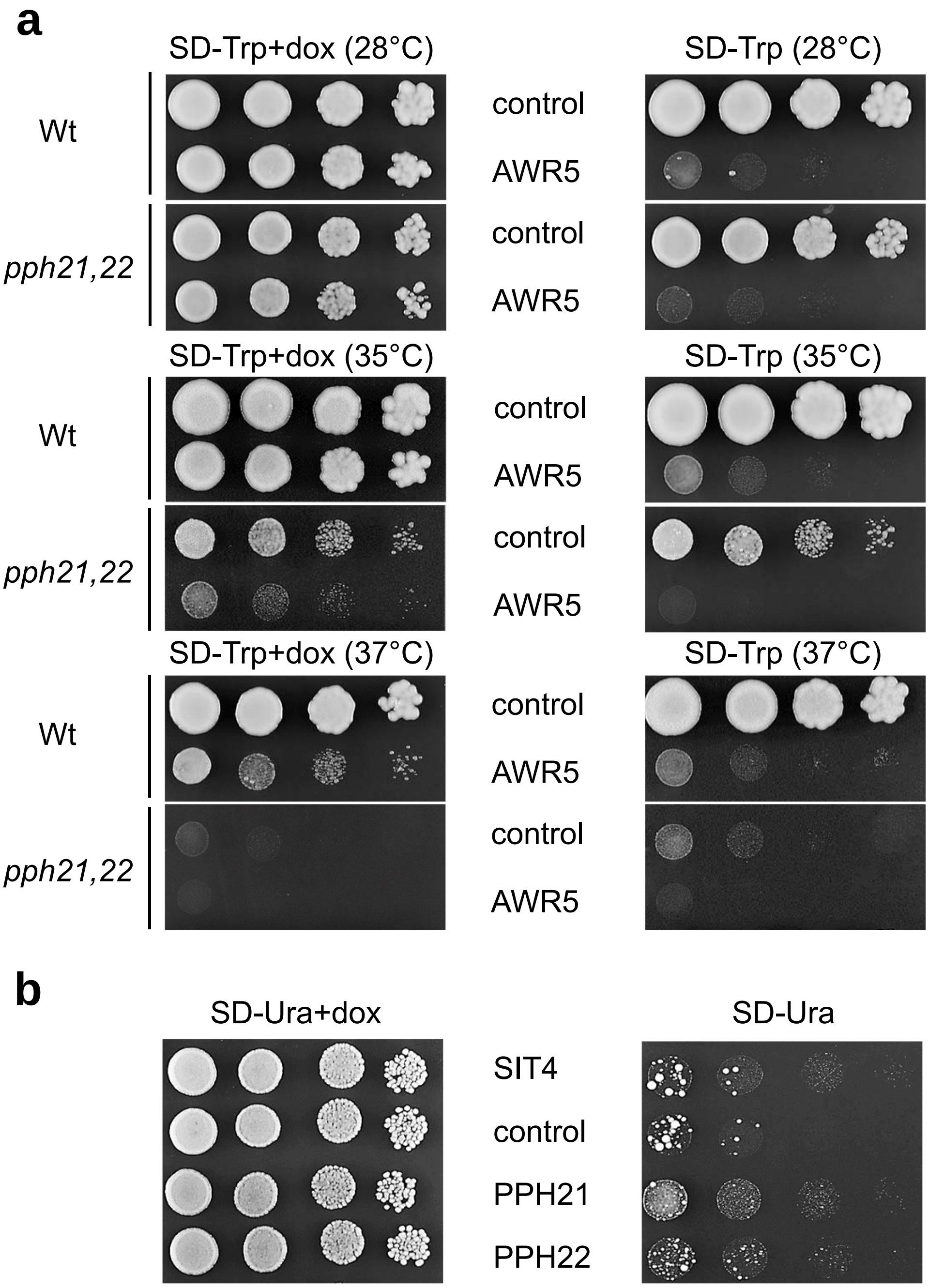

Supplementary Figure S6. Effect of *awr5* expression on yeast strains with altered levels of TORC1-regulated genes. (A) Growth of a wild-type (Wt) and a temperature-conditional *pph21, 22* double mutant expressing *awr5* or carrying an empty vector. Serial 10-fold dilutions were spotted onto solid SD-Trp+doxycycline (repressing medium) and SD-Trp (inducing medium) and plates were incubated at different temperatures (28, 35 and 37°C). (B) Growth of yeast strains expressing *awr5* and overexpressing different TORC1-regulated genes. Serial 10-fold dilutions were spotted onto solid SD-Ura+doxycycline (repression of *awr5*) and SD-Ura (*awr5* expression). All photographs were taken after 3 days of growth.

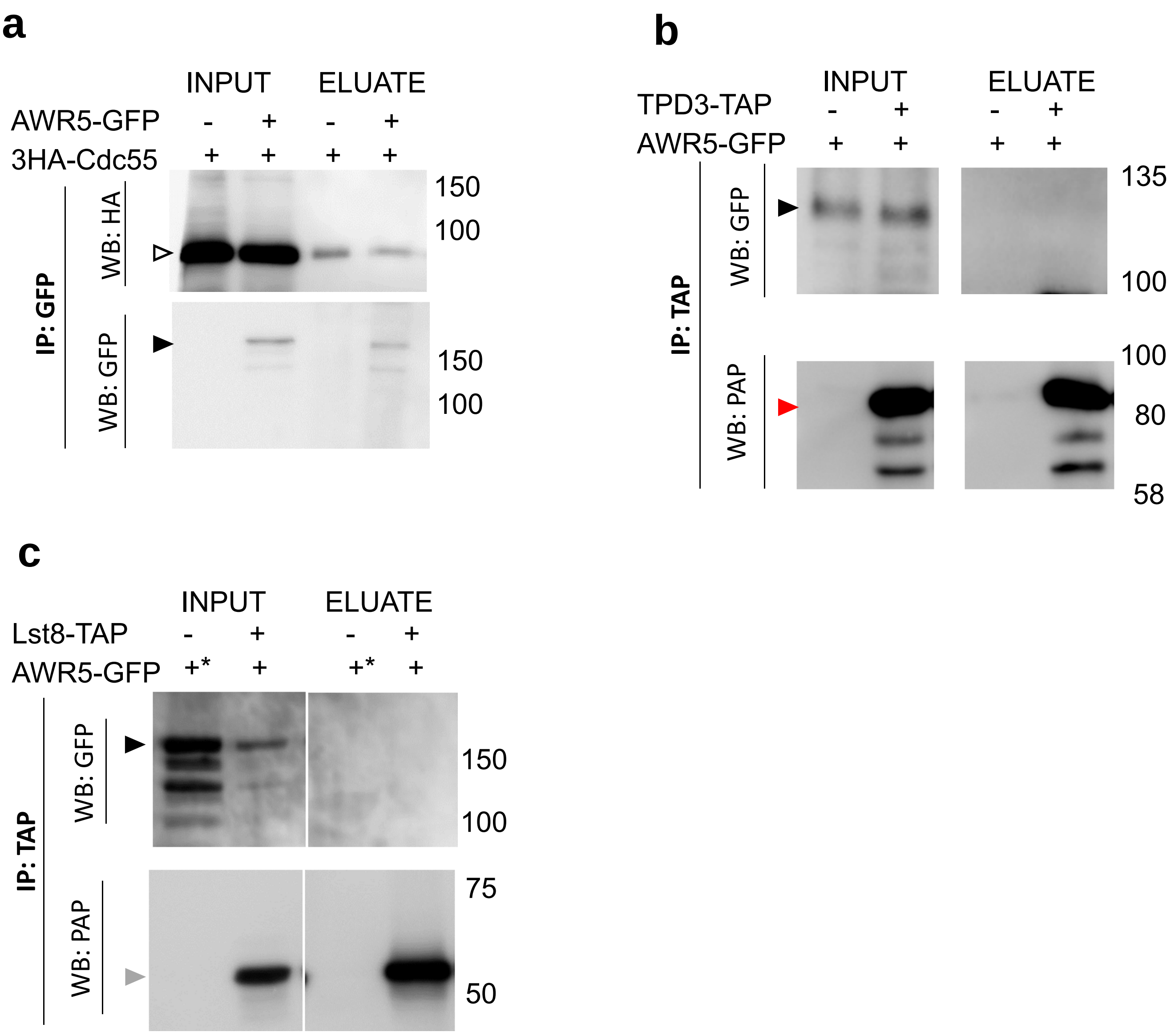

Supplementary Figure S7. AWR5 does not interact with TORC1 signalling components Cdc55, Tpd3 and Lst8 in yeast. (a) Total protein was extracted from yeast expressing 3HA-Cdc55 alone or in combination with AWR5-GFP and immunoprecipitated using magnetic GFP beads. Total (INPUT) or immunoprecipitated (ELUATE) proteins were immunoblotted using anti-HA (top panel) or anti-GFP (bottom panel) antibodies. (b and c) Total protein was extracted from yeast expressing AWR5-GFP alone or in combination with Tpd3-TAP (b) or Lst8-TAP (c) and immunoprcipitated using IgG agarose beads. Total (INPUT) or immunoprecipitated (ELUATE) proteins were immunoblotted using anti-GFP (top panel) or anti-PAP (bottom panel) antibodies. Triangles indicate: 3HA-Cdc55 (white), AWR5-GFP (black), Tpd3-TAP (red) and Lst8-TAP (grey). Numbers on the right indicate molecular weight markers in KDa. Asterisks indicate strains where *awr5* was integrated in the genome. The lack of asterisks denotes strains where *awr5* is plasmid-encoded.

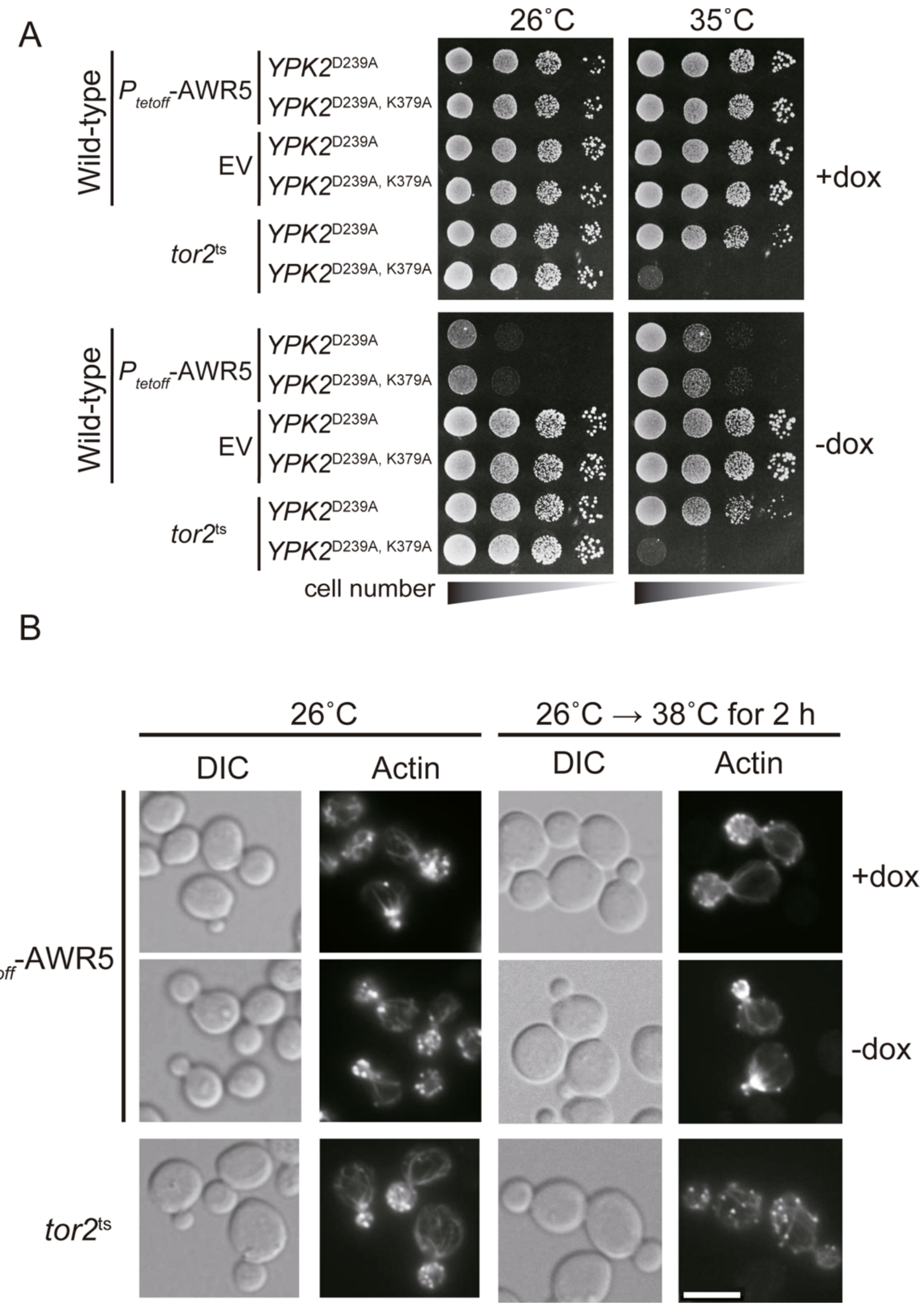

Supplementary Figure S8. AWR5 does not impact on TORC2 pathway in yeast. (a) Expression of dominant active Ypk2 cannot suppress the growth inhibitory effect caused by AWR5 in yeast. Yeast wild-type or tor2^{ts} mutant cells carrying indicated plasmids were spotted on SD (-Ura, -Leu) with or without doxycycline plate and incubated for 3 days at indicated temperature. EV: empty vector. (b) Expression of AWR5 does not impact on actin organization in yeast. Yeast wild-type cells carrying pRS425-Ptetoff-AWR5 plasmid or tor2^{ts} mutant cells were grown in SD (-Leu) with or without doxycycline (20 μg/ml) to early log phase, shifted to the appropriate temperature for 2 hours, fixed and stained with rhodamine-phalloidin. Bar, 5 μm

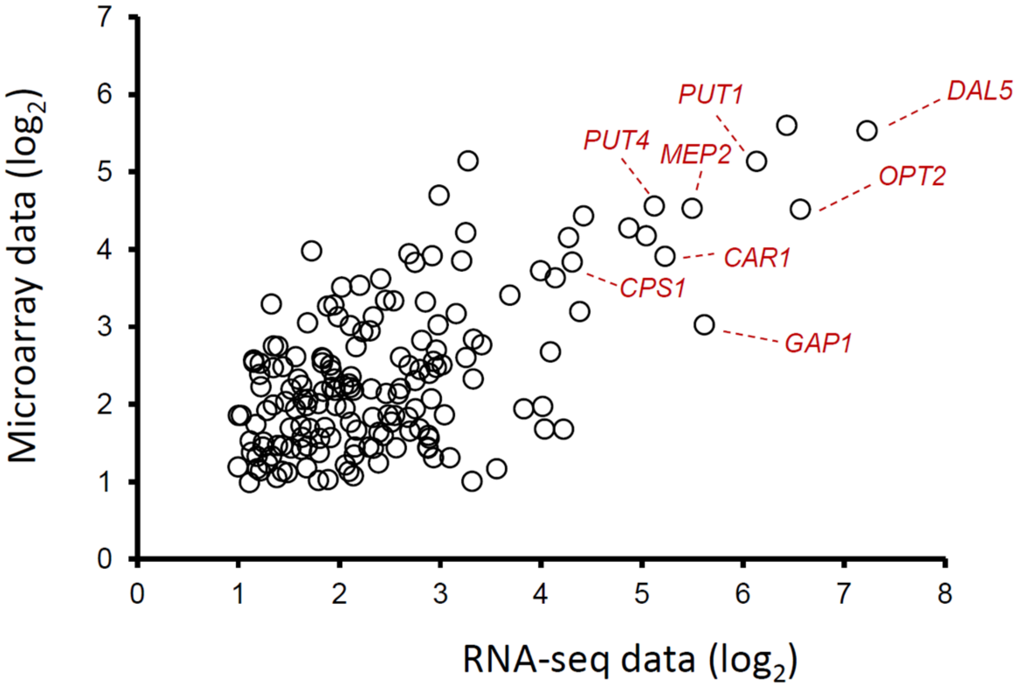

Supplementary Figure S9. Correlation in gene expression data obtained by microarray hibridisation vs RNA-seq. Expression ratios for genes induced genes upon *awr5* expression compared to non-expressing conditions are represented for both methodologies. Key genes mentioned in the text are labelled with their names.

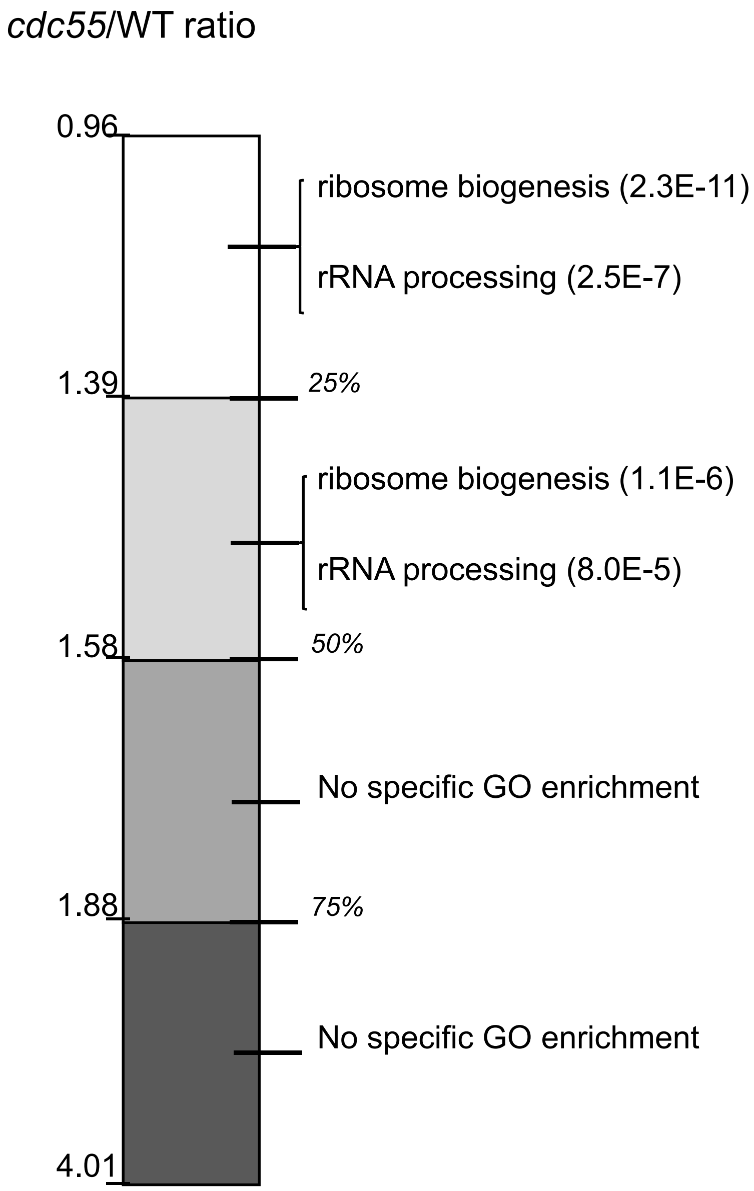

Supplementary Figure S10. Ratio between the repression caused by AWR5 expression in the wild type and *cdc55* strains. The ratio was calculated for 219 genes found to be repressed in the wild type strain. Genes were ranked according to this ratio and the rank divided into quartiles. Gene Ontology analysis was performed for each quartile using the SGD YeastMine tool and relevant results are shown on the right. p-values are shown in parentheses.

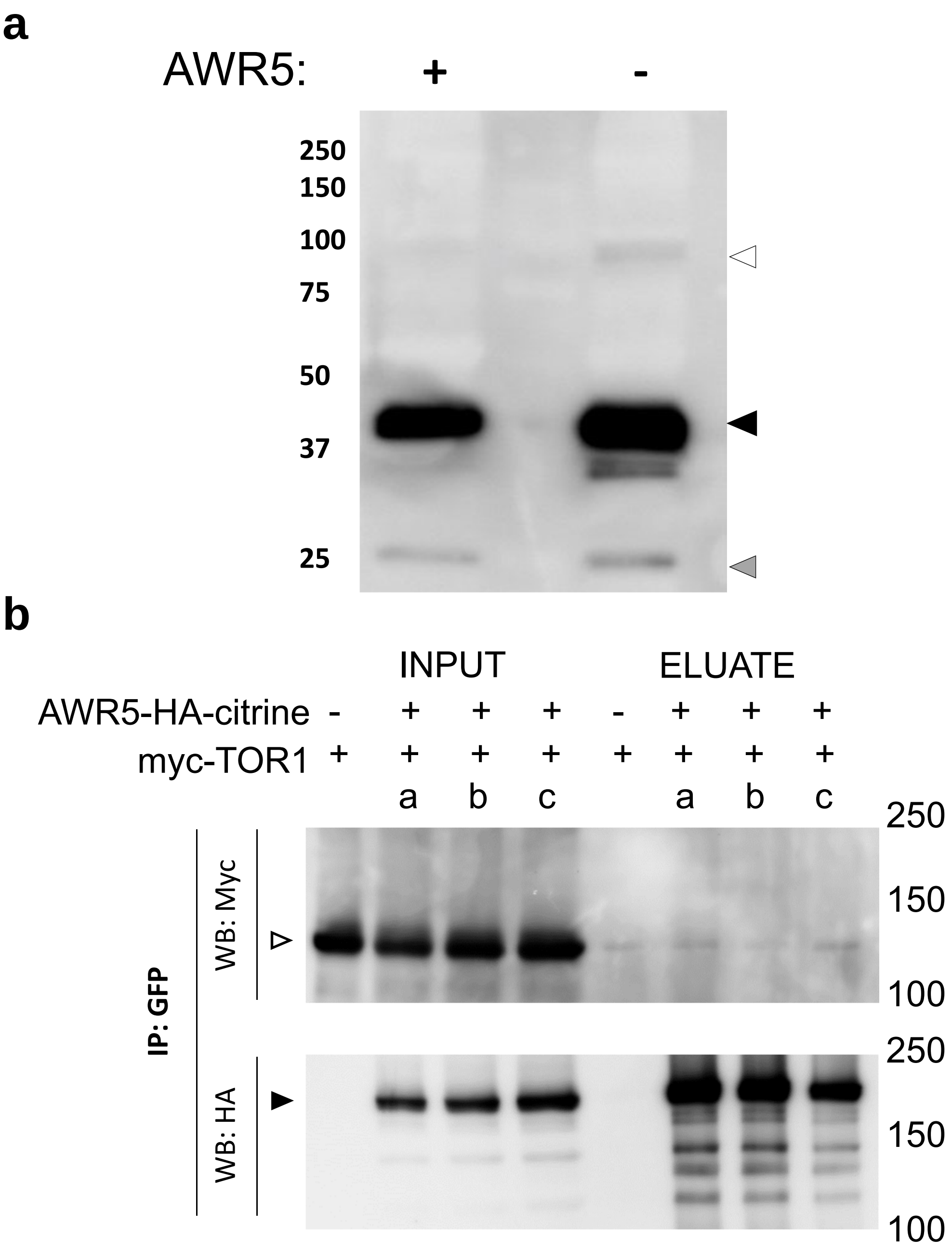

Supplementary Figure S11. Effects of AWR5 expression on autophagy in plants and interaction with TOR. (A) Total protein extracts of *N. benthamiana* leaves transiently expressing the autophagy marker *GFP-ATG8a* alone or in combination with AWR5-citrine-HA were subjected to immunoblot analysis using an anti-GFP antibody. White triangle: AWR5-HA-citrine; black triangle: GFP-ATG8a, grey triangle: GFP. (B) Total protein was extracted from *N. benthamiana* leaves transiently expressing *myc-Tor1* alone or in combination with *AWR5-HA-citrine* and immunoprecipitated using magnetic GFP beads. Total (INPUT) or immunoprecipitated (ELUATE) proteins were immunoblotted using anti-Myc (top panel) or anti-HA (bottom panel) antibodies. Triangles indicate: Myc-Tor1 (white) and AWR5-GFP (black). Numbers on the right indicate molecular weight markers in kDa. Letters (a, b and c) indicate three independent samples extracted from different plants.

TOR RNAi Wild-type *b55α*

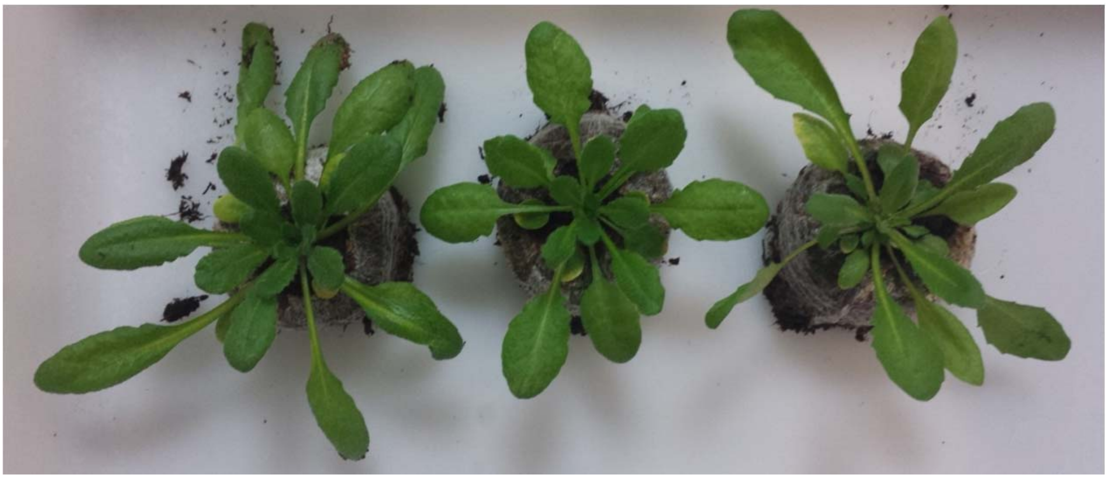

Supplementary Figure S12. Macroscopic phenotype of Arabidopsis lines altered in TOR or b55 genes. Adult 5-week-old TOR RNAi and *b55α* mutant plants used for pathogenicity assays where photographed 3 weeks post transplantation. Notice that altered lines do not show any phenotypical difference compared to the wild-type.
